# A CRISPR-Cas12a integrated SERS nanoplatform with chimeric DNA/RNA hairpin guide for ultrasensitive nucleic acid detection

**DOI:** 10.7150/thno.75816

**Published:** 2022-08-08

**Authors:** Bohan Yin, Qin Zhang, Xinyue Xia, Chuanqi Li, Willis Kwun Hei Ho, Jiaxiang Yan, Yingying Huang, Honglian Wu, Pui Wang, Changqing Yi, Jianhua Hao, Jianfang Wang, Honglin Chen, Siu Hong Dexter Wong, Mo Yang

**Affiliations:** 1 Department of Biomedical Engineering, The Hong Kong Polytechnic University, Kowloon, Hong Kong 999077, China.; 2 Department of Physics, The Chinese University of Hong Kong, Shatin, Hong Kong 999077, China.; 3 Department of Microbiology, The University of Hong Kong, Pokfulam, Hong Kong 999077, China.; 4 Key Laboratory of Sensing Technology and Biomedical Instruments (Guangdong Province), School of Biomedical Engineering, Sun Yat-Sen University, Guangzhou, 510006, P. R. China.; 5 Department of Applied Physics, The Hong Kong Polytechnic University, Kowloon, Hong Kong 999077, China.; 6 Research Institute for Sports Science and Technology, The Hong Kong Polytechnic University, Kowloon, Hong Kong 999077, China.

**Keywords:** gold nanoparticles, magnetic manipulation, surface-enhanced Raman spectroscopy, CRISPR-Cas12a, nucleic acid detection

## Abstract

**Background:** CRISPR-Cas12a has been integrated with nanomaterial-based optical techniques, such as surface-enhanced Raman scattering (SERS), to formulate a powerful amplification-free nucleic acid detection system. However, nanomaterials impose steric hindrance to limit the accessibility of CRISPR-Cas12a to the narrow gaps (SERS hot spots) among nanoparticles (NPs) for producing a significant change in signals after nucleic acid detection.

**Methods:** To overcome this restriction, we specifically design chimeric DNA/RNA hairpins (displacers) that can be destabilized by activated CRISPR-Cas12a in the presence of target DNA, liberating excessive RNA that can disintegrate a core-satellite nanocluster via toehold-mediated strand displacement for orchestrating a promising “on-off” nucleic acid biosensor. The core-satellite nanocluster comprises a large gold nanoparticle (AuNP) core surrounded by small AuNPs with Raman tags via DNA hybridization as an ultrabright Raman reporter, and its disassembly leads to a drastic decrease of SERS intensity as signal readouts. We further introduce a magnetic core to the large AuNPs that can facilitate their separation from the disassembled nanostructures to suppress the background for improving detection sensitivity.

**Results:** As a proof-of-concept study, our findings showed that the application of displacers was more effective in decreasing the SERS intensity of the system and attained a better limit of detection (LOD, 10 aM) than that by directly using activated CRISPR-Cas12a, with high selectivity and stability for nucleic acid detection. Introducing magnetic-responsive functionality to our system further improves the LOD to 1 aM.

**Conclusion:** Our work not only offers a platform to sensitively and selectively probe nucleic acids without pre-amplification but also provides new insights into the design of the CRISPR-Cas12a/SERS integrated system to resolve the steric hindrance of nanomaterials for constructing biosensors.

## Introduction

Integrating clustered regularly interspaced short palindromic repeats (CRISPR)-associated nuclease (Cas, such as Cas12a) with analytical methods has become prevalent for nucleic acid detection 1-3. CRISPR-Cas12a performs a rapid, efficient, and collateral trans-cleavage of nearby non-specific single-stranded DNA (ssDNA) after the activation by its associated CRISPR RNA (crRNA), forming a duplex with a target DNA. Based on this mechanism, an ssDNA-linked fluorophore-quencher pair can be uncoupled by activated CRISPR-Cas12a to report fluorescence signals upon detecting target DNA 2, 4. To improve the sensitivity, contemporary studies have focused on magnifying the fold change of the signal readouts between the initial and sensing states. For instance, “off-on” detection systems have been designed through the CRISPR-Cas12-mediated new fragments to induce the assembly of nanoparticles (NPs) to form nanoclusters, generating color change or colorimetric signal change as a result 5, 6. However, these strategies often require target pre-amplification to achieve sensitive detection, which may result in additional procedures and increase the risk of false-positive signals.

Different types of biosensing techniques, including fluorescence 7, colorimetric 8, electrochemical 9, and field effect transistor (FET) 10, have been developed for nucleic acid detection. Among various biosensing techniques, surface-enhanced Raman scattering (SERS) has emerged as a promising optical approach due to the advantages of high sensitivity and rapid response 11. For instance, Raman tags are resistant to photobleaching and possess narrow characteristic spectral fingerprints that are highly useful for nucleic acid detection 12. Noble metallic nanostructures (e.g., gold NPs, AuNPs) play an important role in SERS-based biosensors because of their unique optical properties, including the amplification of electromagnetic fields (EMF) when localized surface plasmon resonance (LSPR) is excited 13, 14. To optimize the performance of the SERS-based biosensors, 'hot spots' are generated at nanoscale junctions (< 30 nm) between two closely coupled AuNPs to amplify the SERS intensity of Raman tags with a possible enhancement factor up to 10^14^ 15-17. Recent studies employed CRISPR-Cas12a to mediate the disconnection of SERS-active nanostructures from the detection platform, leading to its attenuated SERS signal for establishing SERS-based “on-off” biosensors 18, 19. To maximize the signal difference between “on” and “off”, Raman tags can be placed at hot spots between two ssDNA-coupled plasmonic nanostructures, which are theoretically separable by activated CRISPR-Cas12a to remove the hot spots. Nevertheless, it has been reported that nanomaterials impose a strong steric hindrance to restrict the accessibility of CRISPR-Cas12a to ssDNA on their surface 20-22, probably due to the large hydrodynamic size of CRISPR-Cas12a (> 100 nm) 23. This steric effect is much higher at the narrow gaps among NPs, further hindering CRISPR-Cas12a from assessing hot spots 24. Hence, it is challenging to manipulate the merits of both SERS and CRISPR-Cas12a to build an ultrasensitive amplification-free platform for probing target DNA. We have previously achieved magnetic control over the gap distance between two layers of plasmonic nanostructures that sandwich the N gene of severe acute respiratory syndrome coronavirus 2 (SARS-CoV-2) for constructing tunable hot spots to improve SERS signals post-detection 25. Nevertheless, few studies have proposed a method to resolve the issue of nanomaterials steric hindrance for accomplishing hot spot removal and maximizing SERS signal changes of CRISPR-Cas12a/SERS-based “on-off” systems toward the sensitive detection of target analytes.

**Figure 1:**
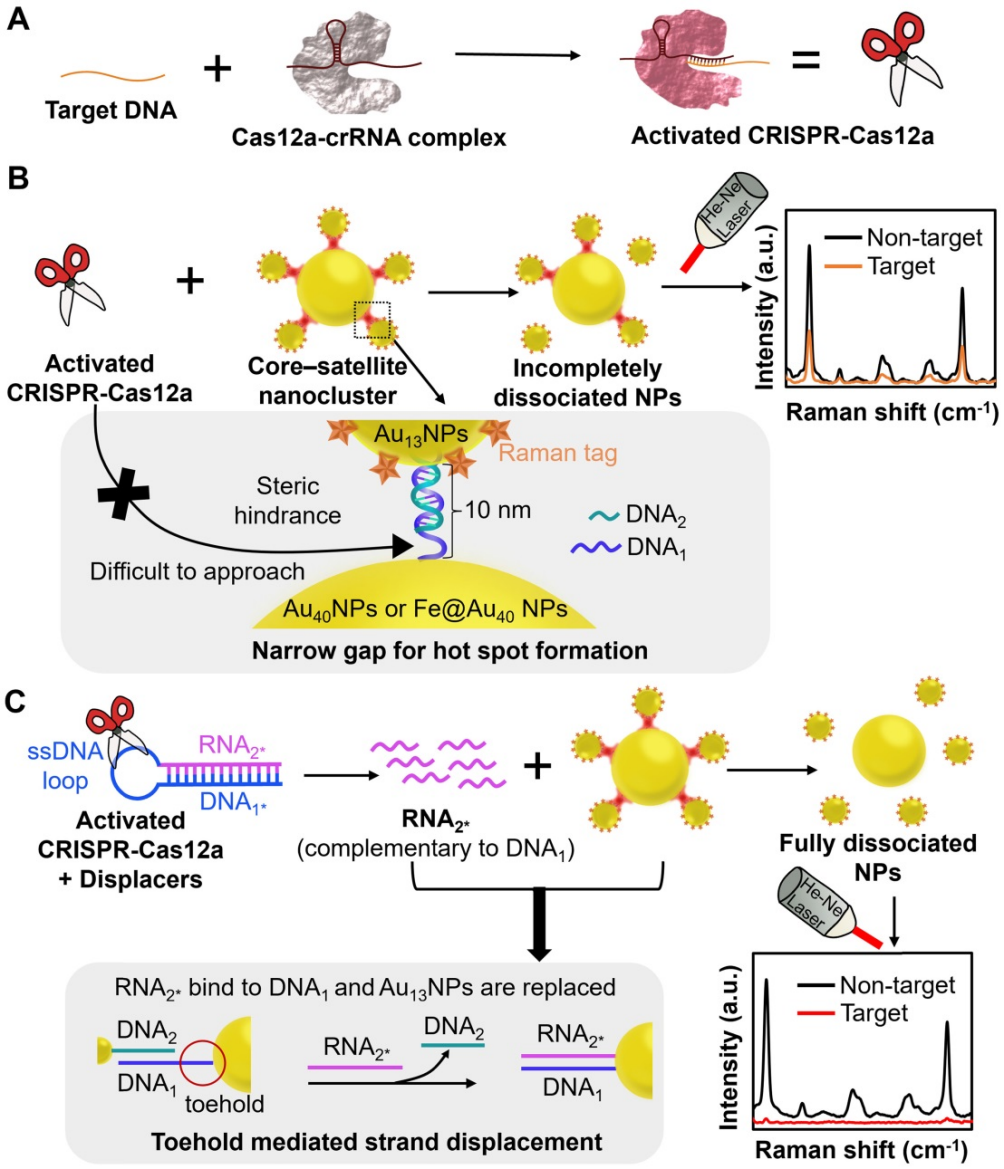
** The working principle of the CRISPR-Cas12a/SERS integrated biosensing system with or without the introduction of the displacer.** (A) In the presence of target DNA, the activated CRISPR-Cas12a/crRNA complex immediately performs trans-cleavage. Core-satellite nanoclusters were fabricated as the initially “on” ultrabright Raman reporter. It consists of DNA_1_-Au_40_NPs or DNA_1_-Fe@Au_40_ NPs as the core, and MBA/DNA_2_-Au_13_NPs as the surrounding satellites linked by a partially complementary DNA pair (DNA_1_/DNA_2_). (B) Without the displacer, steric hindrance imposed by the coupled AuNPs limited the accessibility of the activated CRISPR-Cas12a to the ssDNA at the narrow gaps, leading to incomplete dissociation of the nanoclusters and high SERS signal after detecting target DNA. (C) With the displacer, the activated CRISPR-Cas12a cleaves the ssDNA hairpin loop of the displacer composed of completely hybridized DNA_1*_ and RNA_2*_, liberating a large number of RNA_2*_. Note that RNA_2*_ is entirely complementary to DNA_1_, and DNA_1*_ has the same sequence as DNA_1_. The pre-treated displacer (released RNA_2*_) was mixed with the core-satellite nanoclusters in the hybridization buffer and bound to the toehold of DNA_1_ on Au_40_NPs or Fe@Au_40_ NPs, resulting in fully dissociated NPs and low SERS signal after detecting target DNA.

In this work, we specifically design a chimeric DNA/RNA hairpin and introduce it into CRISPR-Cas12a/SERS integrated system to overcome the steric effect for ultrasensitive, highly selective, and amplification-free nucleic acid detection (Figure 1). Notably, we exploit ssRNA released from the chimeric DNA/RNA hairpin to disintegrate nanoclusters instead of using CRISPR-Cas12a directly to avoid the strong steric hindrance imposed by the nanoclusters. As the single-stranded nucleic acid is known to be small in size and flexible 26, it has been employed to access the gaps among NPs to disassemble the nanoclusters through toehold-mediated strand displacement reaction (TMSDR) 27, 28. Our sensing system comprises two main components: 1) an initially “on” ultrabright Raman reporter, core-satellite nanoclusters consisting of 40 nm AuNPs (DNA_1_-Au_40_NPs) as the core, and Raman tag (4-mercaptobenzoic acid; MBA)-coated 13 nm AuNPs (MBA/DNA_2_-Au_13_NPs) as the surrounding satellites linked by a partially complementary DNA pair (DNA_1_/DNA_2_) (Table S1); and 2) a CRISPR-Cas12a with a chimeric DNA/RNA hairpin (displacer) that contains a nucleic acid with stem-loop structure where the stem is formed by fully hybridized DNA_1*_ and RNA_2*_, and the loop is an A-T repeating DNA sequence (Table S1). In the presence of target DNA, CRISPR-Cas12a is activated to immediately cleave the DNA loops of displacers, releasing a large number of RNA_2*_ (Figure 1) 6. The released RNA_2*_ is entirely complementary to DNA_1_ and hence is energetically favourable to replace DNA_2_ and hybridize with DNA_1_ on Au_40_NPs through TMSDR 27, 29, 30, leading to the detachment of MBA/DNA_2_-Au_13_NPs from DNA_1_-Au_40_NPs for hot spot removal and the sequential decrease of SERS signal of the sensing platform (“on-off”). We select the Au_13_NP-Au_40_NP pair to form core-satellite nanoclusters because AuNPs larger than 60 nm can easily sediment that may cause agglomeration and are not suitable for long-term storage 31, and Au_13_NPs can be prepared by the one-step synthesis 32. In this proof-of-concept study, the CRISPR-Cas12a-treated displacer group was ~3.5 fold more effective (in the presence of 1 nM target DNA) in decreasing the SERS intensity of the system and attained a better limit of detection (LOD, 10 aM) than those by only employing activated CRISPR-Cas12a for target DNA detection. To further improve the LOD of our sensing platform, we replace the Au_40_NPs with Au-coated superparamagnetic iron oxide NPs with a diameter of ~40 nm (Fe@Au_40_ NPs) 33. The large core AuNPs are magnetically isolated from the disassembled core-satellite nanoclusters, significantly suppressing the background signals and resulting in improved LOD at 1 aM (~600 copies/mL). Furthermore, we prove the repeatability and reliability of our system by the small values of percent relative standard deviation (%RSD) and recovery values close to 100%, respectively, upon detecting target DNA at defined concentrations in various buffers or relevant biological fluids. To the best of our knowledge, limited studies have manifested the special design of chimeric DNA/RNA hairpins and trans-cleavage activity of CRISPR-Cas12 to optimize a SERS-based “on-off” system for quantitative nucleic acid detection.

## Results and Discussion

### Characterization and optimization of core-satellite nanoclusters with multiple hot spots as a strong Raman reporter in this biosensing system

We first fabricated core-satellite nanoclusters as the initially “on” ultrabright Raman reporter in the biosensing system. DNA_1_-Au_40_NPs and MBA/DNA_2_-Au_13_NPs were synthesized and characterized by transmission electron microscope (TEM) for their anhydrous sizes and DNA shells (Figure S1A-C; Figure S2A-C), ultraviolet-visible (UV-vis) spectrophotometer for their characteristic SPR peaks (Figure S1D; Figure S2D), and dynamic light scattering (DLS) for their hydrodynamic sizes and zeta potentials (Figure S1E; Figure S2F). The MBA molecules were loaded onto the vacancies of DNA_2_-Au_13_NP surface 25, exhibiting an enhanced Raman signal compared to that of MBA molecules only (Figure S2E). We picked the peak of MBA at 1078 cm^-1^, corresponding to its ring stretch vibration mode 34, as the characteristic peak (MBA-peak) for signal readout calculation in this study. The optimal hybridization molar ratio of DNA_1_-Au_40_NPs and MBA/DNA_2_-Au_13_NPs was 1:5, as the MBA-peak intensity of the hybridized NPs at this ratio reached a plateau while the addition of more MBA/DNA_2_-Au_13_NPs (e.g., ratio of 1:7.5 or 1:10) contained excessive unhybridized MBA/DNA_2_-Au_13_NPs that might increase the background noise during target DNA detection (Figure S3). Considering that shortening the DNA pair for Au_40_NPs and Au_13_NPs linkage may lead to stronger hot spot formation, we designed a half-length DNA pair of the original one (DNA_1HL_/DNA_2HL_, Table S1). However, after incubating DNA_1HL_-Au_40_NPs and MBA/DNA_2HL_-Au_13_NPs at the optimal hybridization molar ratio (1:5), the SERS signal of the core-satellite nanoclusters only exhibited 35.12% of that of the core-satellite nanoclusters linked by DNA_1_/DNA_2_ (Figure S4). This shortened pair did not have the privilege of producing a stronger SERS signal in the core-satellite nanoclusters, probably due to the less stable binding of Au_40_NPs and Au_13_NPs via DNA_1HL_/DNA_2HL_. Therefore, DNA_1_/DNA_2_ is optimal for constructing the ultrabright Raman reporter in our biosensing platform.

The core-satellite nanoclusters of DNA_1_-Au_40_NPs or MBA/DNA_2_-Au_13_NPs showed a redshift of their SPR peak and a larger hydrodynamic size compared to DNA_1_-Au_40_NPs or MBA/DNA_2_-Au_13_NPs only (Figure S5A-C,E,G). The estimated gap distance between DNA_1_-Au_40_NPs and MBA/DNA_2_-Au_13_NPs was controlled at ~10 nm, according to the theoretical length of hybridized DNA_1_ and DNA_2_ 35, 36. This close interparticle distance ascertains the formation of hot spots that amplify the SERS intensity, as evidenced by the significantly elevated MBA-peak intensity of the core-satellite nanoclusters (~27-fold increase) than that of MBA/DNA_2_-Au_13_NPs alone (Figure S5F). Consistently, our simplified simulation using finite-difference time-domain (FDTD) method illustrates a representative hot spot in the core-satellite nanoclusters, revealing a pronouncedly enhanced electric field in the hot spot region when compared to a single MBA/DNA_2_-Au_13_NP (Figure S5D). On the contrary, the MBA-peak intensity of a mixture of DNA_1_-Au_40_NPs and MBA/DNA_2_-Au_13_NPs without hybridization buffer exhibited only ~7% of that of the core-satellite nanoclusters, further confirming that the close interparticle distance was achieved by DNA hybridization (Figure S6F-G). To create a positive control as “off” condition, we dissociated the core-satellite nanoclusters by incubation at 65 °C (higher than its melting temperature) for 30 min and observed a decrease of MBA-peak intensity by ~85% compared to that of the intact one (Figure S6F-G). Besides, those dissociated NPs showed a blueshift of SPR peak with a smaller hydrodynamic size compared to the core-satellite nanoclusters (Figure S6A-E; Table S2). These results confirm that MBA/DNA_2_-Au_13_NPs closely bind to DNA_1_-Au_40_NPs for hot spot formation, and this system can be dissociated for hot spot removal. Moreover, we verified that the core-satellite nanoclusters exhibited minimal SERS signal variations over 14 days of storage at 4 oC, confirming their good stability for storage (Figure S7).

In short, we have successfully built a strong, stable, and well-characterized Raman reporter with multiple SERS hot spots.

### Optimization of the length of the displacer for disintegrating core-satellite nanoclusters in the presence of the target nucleic acid

Displacers of 3 different lengths were investigated to examine TMSDR effectiveness between the released RNA and DNA_1_/DNA_2_ duplex. By the number of nucleotides (nt) in the stem (RNA) of the displacer for binding to the toehold of DNA_1_, the displacers can be classified into 1-nt displacer, 3-nt displacer, and 6-nt displacer. We first performed a thermodynamic calculation to examine the feasibility of TMSDR mediated by these 3 types of displacers (Figure S8), according to a previous report 27. Significantly, our calculation results illustrate that 6-nt displacer is the most energetically favourable with standard Gibbs free energy change ΔG° < 0 and the equilibrium constant K > 1 in this TMSDR among these 3 types of displacers (Figure S8).

We next proved the feasibility of the liberation of RNA_2*_ from DNA_1*_ in the stem of 6-nt displacer after the treatment of target-activated CRISPR-Cas12a. We designed a DNA/RNA chimeric hairpin-based molecular beacon (F-displacer-D) that has the same sequence as the 6-nt displacer in our detection system but is modified with a fluorophore (FAM) on the 5' of RNA_2*_ and a quencher (DABCYL) on 3' of DNA_1*_ (Table S1). This as-quenched structure initially showed a limited fluorescence signal via Förster resonance energy transfer (FRET) (Figure S9). Our data demonstrate that the F-displacer-D treated by target-activated CRISPR-Cas12a largely recovered the fluorescence intensity compared to its equivalent amount of counterpart (single-stranded RNA with a fluorophore, FAM-RNA_2_). In contrast, the F-displacer-D treated by CRISPR-Cas12a in the presence of a scrambled version of the target or blank samples remained with minimal fluorescence signal (Figure S9). It is because cleavage of the displacer loop changes the nature of hybridization in the stem region from intra- to inter-molecular interaction that destabilizes the hybrid and liberates RNA_2*_ 6. These results confirm that target-activated CRISPR-Cas12a could effectively mediate the release of RNA_2*_ from the treated chimeric DNA/RNA hairpin.

To optimize the reaction time for displacer-mediated disassembly of the core-satellite nanoclusters via TMSDR, we directly administrated excessive RNA_2_ sequence (~1.5 folds and ~2.4 folds of DNA_1_ and DNA_2_, respectively) to the core-satellite nanoclusters for different durations. The SERS intensity significantly decreased by 19.09%, 54.98%, and 99.06% after 0.5 h, 1 h, and 2 h of incubation, respectively (Figure S10). Note that there was no significant difference in the SERS intensity between 2-h and 3-h incubation groups. These results indicate that the optimal incubation time for the displacement was 2 h. In contrast, the SERS intensity of the core-satellite nanoclusters remained unchanged in the presence of both the scrambled version of RNA_2_ (Scr-RNA_2_) or displacer only (Figure S10). It also indicates that the 6-nt displacer is sufficiently stable in the absence of activated CRISPR-Cas12a to prevent non-specific disturbance of the core-satellite nanoclusters.

Furthermore, we employed the gel electrophoresis analysis to assess the feasibility of TMSDR mediated by all 3 types of displacers. Before the TMSDR, the displacers (all 3 types) that were treated by target-activated CRISPR-Cas12a appeared as a dimmer and shifted band compared to the intact displacer band, indicating the successful cleavage of the loop and the decrease of DNA/RNA duplex concentration (Figure S11; Figure S12). After the incubation of the 6-nt displacer that was treated by target-activated CRISPR-Cas12a and DNA_1_/DNA_2_ hybrid solution, the gel electrophoresis image reveals the appearance of DNA_2_ band, indicating that DNA_2_ was released by the treated displacer from DNA_1_/DNA_2_ hybrid via TMSDR (Figure S13). In contrast, incubation of DNA_1_/DNA_2_ hybrid with 1-nt or 3-nt displacer that was treated by target-activated CRISPR-Cas12a failed to release a visible DNA_2_ band from the DNA_1_/DNA_2_ hybrid (Figure S14). Besides, the control groups containing displacers that were treated by non-activated CRISPR-Cas12a and DNA_1_/DNA_2_ hybrid did not exhibit this band either (Figure S13; Figure S14). These gel electrophoresis data are in line with the thermodynamic calculation results, further confirming the feasibility of our TMSDR between released RNA_2*_ from 6-nt displacer and DNA_1_/DNA_2_ duplex (Figure S8). These results also prove that the TMSDR is highly specific and efficient with minimal reversible reaction, which is up to 6 orders of magnitude slower than that of the displacement process, according to the literature 37. Since the 6-nt displacer is optimal for the TMSDR, we applied 6-nt displacer (denoted as displacer) throughout the rest of our study.

Overall, we postulate that the SERS intensity of the Raman reporter can be prominently suppressed as the signal readout (“on-off”) by CRISPR-Cas12a/displacer-mediated removal of the hot spots via TMSDR in the presence of target DNA.

### A pronouncedly decreased signal of the CRISPR-Cas12a/SERS integrated system upon nucleic acid detection achieved by the introduction of displacers

**Figure 2:**
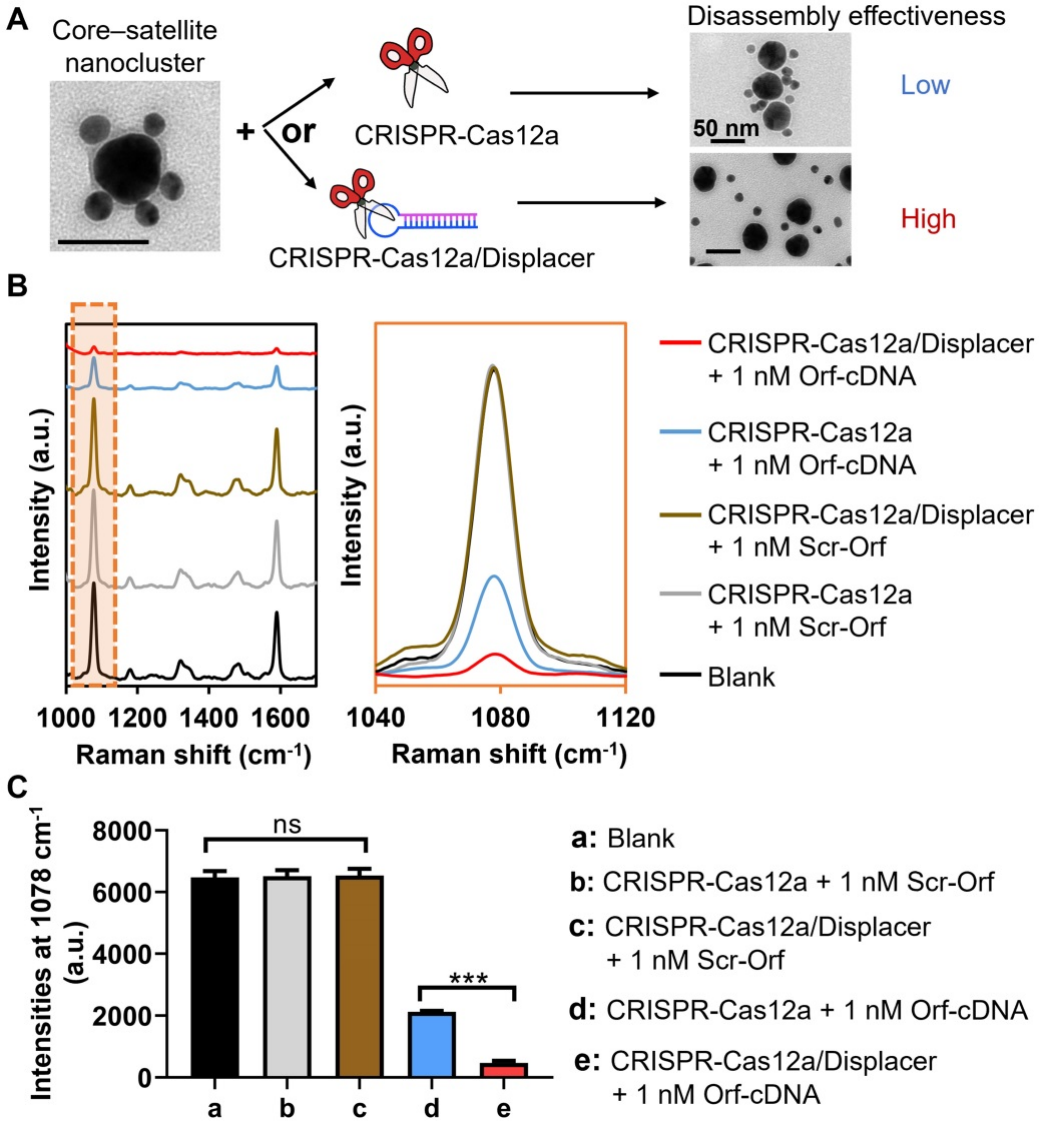
**Evaluation of detection performance of the biosensing system by the CRISPR-Cas12a-based method with (CRISPR-Cas12/Displacer) or without displacer (CRISPR-Cas12a) for detecting Orf-cDNA.** (A) Illustration by representative TEM images of directly employing CRISPR-Cas12a (upper panel with low disassembly effectiveness) for disassembling core-satellite nanoclusters compared to using CRISPR-Cas12a-treated displacers (CRISPR-Cas12a/Displacer, lower panel with high disassembly effectiveness). The scale bar is 50 nm. (B) SERS spectra of biosensing platform after detecting blank samples, 1 nM Scr-Orf, and 1 nM Orf-cDNA. SERS spectra were zoomed into the region of 1040 to 1120 cm^-1^ to compare MBA-peak intensities between CRISPR-Cas12a group and CRISPR-Cas12a/Displacer group in the presence of Orf-cDNA at 1 nM. (C) MBA-peak intensities of the biosensing platform after detection of samples, corresponding to (B). Error bar denotes the standard deviation resulting from three independent experiments. Statistical analysis of pairwise comparison was determined by one-way ANOVA. No significance (ns): P > 0.05; *** P < 0.001.

To prove our hypothesis that the introduction of displacers can overcome the limitation of CRISPR-Cas12a in SERS-based applications, we compared the effectiveness of hot spot removal by CRISPR-Cas12a with that by CRISPR-Cas12a/displacer (Figure 2). In this proof-of-concept study, we select Orf gene (Orf-cDNA) of SARS-CoV-2 as the target DNA, which has been investigated in our previous reports 38. Expectedly, CRISPR-Cas12a only triggered a 67% decrease of the MBA-peak intensity of the system in the presence of 1 nM Orf-cDNA, and the resultant signal was ~3.5-fold higher than that caused by CRISPR-Cas12a/displacer-mediated dissociation (Figure 2B-C). Also, the CRISPR-Cas12a/displacer group showed more singly dispersed cores and satellites, while more incompletely disassembled core-satellite nanoclusters remained in the CRISPR-Cas12a only group (Figure 2A). These findings suggest the low effectiveness of hot spot removal by CRISPR-Cas12a at the nanoscale gaps among NPs. Importantly, our data indicate that the displacer plays an essential role in disintegrating DNA hybridization-coupled metallic nanostructures for constructing a promising “on-off” biosensing system throughout the study.

**Figure 3:**
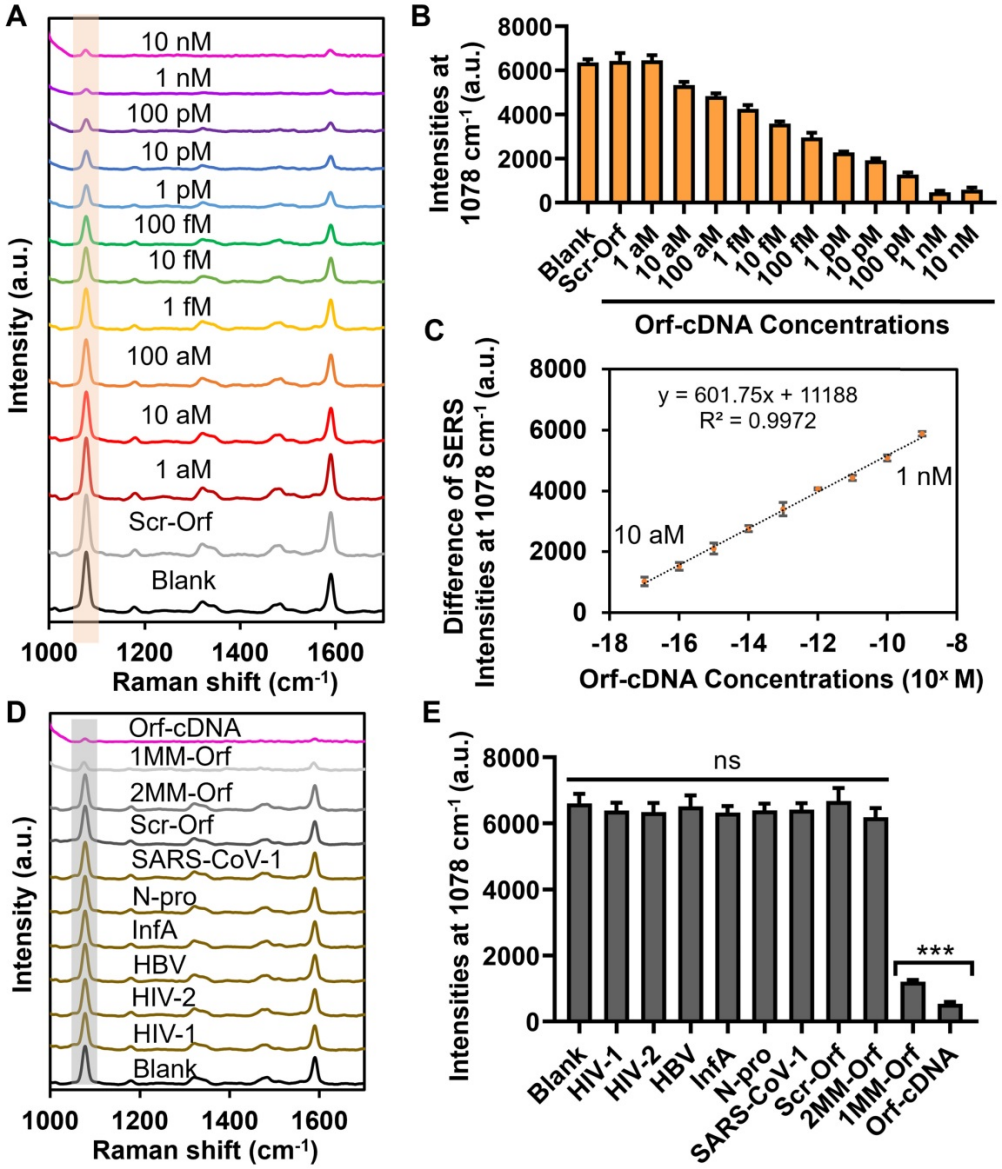
** Sensitivity and selectivity studies of biosensing platform for detecting Orf-cDNA.** (A) SERS spectra of the biosensing platform after detection of blank samples, Scr-Orf (1 nM), and Orf-cDNA at varying concentrations (from 1 aM to 10 nM), displayed from bottom to top. (B) MBA-peak intensities of the biosensing platform after detection of samples, corresponding to (A). (C) Linear relationship of the decrease of MBA-peak intensities and the logarithm (log) concentrations of Orf-cDNA from 10 aM to 1 nM. (D) SERS spectra of the biosensing platform after detection of blank samples, nontarget sequences [cDNA of human immunodeficiency virus type 1 (HIV-1), human immunodeficiency virus type 2 (HIV-2), DNA sequence of hepatitis B virus (HBV), cDNA of influenza A virus (InfA), nucleocapsid (N) gene of SARS-CoV-2 (N-pro), and N gene of SARS-CoV-1 (SARS-CoV-1)], Scr-Orf, 2MM-Orf, 1MM-Orf, or Orf-cDNA, displayed from bottom to top. (E) MBA-peak intensities of the biosensing platform after detection of samples, corresponding to (D). Error bar denotes the standard deviation resulting from three independent experiments. Statistical analysis of pairwise comparison was determined by one-way ANOVA. No significance (ns): P > 0.05; *** P < 0.001.

We subsequently evaluated the detection sensitivity of the displacer-guided platform by analyzing the SERS spectra of our platform toward target DNA with concentrations ranging from 1 aM to 10 nM. Strikingly, the decrease in MBA-peak intensity was significant when the concentration of target DNA was higher than 10 aM, and the decline reached a plateau with the target concentration ≥ 1 nM. Moreover, our sensing results indicate a linear relationship between the difference in MBA-peak intensity (subtracted from signals of blank samples) and logarithm (log) concentrations of Orf-cDNA from 10 aM to 1 nM (R^2^ = 0.9972) with a LOD at 10 aM (Figure 3A-C; Table S3). Likewise, the TEM images confirm the dissociation of the core-satellite nanoclusters by CRISPR-Cas12-treated displacers after detecting Orf-cDNA at 1 nM rather than blank samples or a scrambled version of Orf-cDNA (Scr-Orf) at 1 nM (Figure S15). For the signal measurement, we examined 3 independent samples and collected SERS signals of 6 random points from each sample. The %RSD of MBA-peak intensities for 1 nM or 10 aM Orf-cDNA group is 9.87% or 2.66%, respectively, indicating good repeatability of the displacer-based assay (Figure S16). On the other hand, the CRISPR-Cas12a only group was unable to attain the LOD at 10 aM for sensing target DNA in this sensing system (Figure S17), consistent with its low disassembly effectiveness and indicating the limitation of integrating CRISPR-Cas12a with SERS-active nanoarchitectures for sensitive nucleic acid detection.

### Selectivity study of the biosensing system

We next evaluated the detection selectivity of the displacer-guided biosensing system by including Scr-Orf together with 6 different types of viral sequences as other control (nontarget) groups (Figure S18B; Table S1). Base-mismatched (single- or double-mismatched) sequences of Orf-cDNA (1MM- or 2MM-Orf) served to emulate the mutation of SARS-CoV-2 genes (Figure S18A; Table S1) 39. By gel electrophoresis, the addition of scrambled or nontarget sequences did not mediate the formation of a new band (Figure S11), indicating that our CRISPR-Cas12a system was highly specific to Orf-cDNA. Consistently, the overall SERS intensities after detecting nontarget DNA sequences at a concentration of 1 nM exhibited no significant difference from that of blank samples (Figure 3D-E). Also, the MBA-peak intensity of 1MM-Orf group was 2-fold as much as that of Orf-cDNA group, and the MBA-peak intensity of 2MM-Orf group was similar to that of the nontargets at the same DNA concentration. These results indicate that this displacer-integrated SERS platform was able to distinguish the difference between target DNA and nontarget/base-mismatched DNA sequences, thereby being suitable for selective nucleic acid detection.

### Improvement of the biosensing sensitivity by magnetic separation

**Figure 4:**
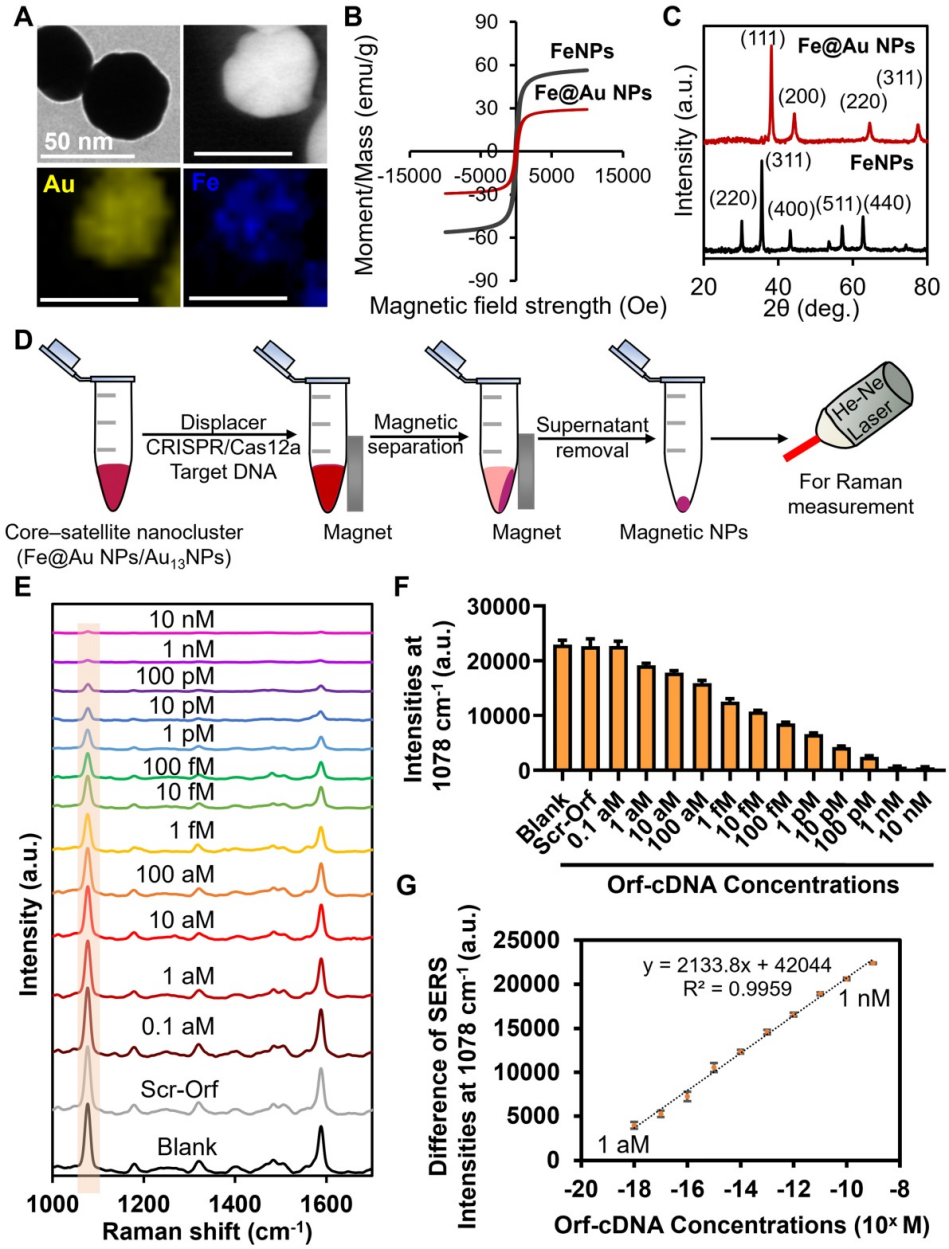
**Sensitivity study of detecting Orf-cDNA using the magnetic-responsive biosensing platform.** (A) Representative TEM image, scanning transmission electron microscope (STEM) image, and EDX elemental map of Fe@Au_40_ NPs. (B) By vibrating-sample magnetometer (VSM) measurement, the magnetic moment per unit mass of FeNPs and Fe@Au_40_ NPs exhibited minimal hysteresis, and their saturation moments (M_sat_) were estimated to be 56 emu/g and 29 emu/g, respectively. (C) Typical XRD patterns of FeNPs and Fe@Au_40_ NPs, respectively. (D) Schematic illustration of magnetic reduction of background signal after detection of target DNA. Dissociated MBA/DNA_2_-Au_13_NPs without magnetic property were removed after the magnetic separation. (E) SERS spectra of the biosensing platform after detection of blank samples, Scr-Orf (1 nM), and Orf-cDNA at varying concentrations (from 0.1 aM to 10 nM), displayed from bottom to top. (F) MBA-peak intensities of the biosensing platform after detecting samples, corresponding to (E). (G) Linear relationship of the decrease of MBA-peak intensities and the log concentrations of Orf-cDNA from 1 aM to 1 nM. Error bar denotes the standard deviation resulting from three independent experiments.

Despite the fact that displacer-mediated hot spot removal in the core-satellite nanoclusters was effective for sensitive and selective detection of target DNA, we question whether the LOD of this biosensing system can be further improved. Previous studies applied superparamagnetic iron oxide nanoparticles (FeNPs) for nucleic acid extraction, target enrichment, and infectious disease diagnosis because of their attractive magnetic properties at the nanoscale 40. Thus, we replaced the Au_40_NPs with Fe@Au_40_ NPs (~40 nm) to construct DNA_1_-Fe@Au_40_ NPs and facilitate their separation from MBA/DNA_2_-Au_13_NPs, further decreasing the background signal post-detection (Figure 4A-C; Figure S19; Figure S20; Figure S21). Hence, we first investigated the SERS intensity of the magnetically separated nanoclusters (10-min magnetic attraction) from the dissociated core-satellite nanoclusters after incubation at 65 °C. Notably, we observed that the magnetic separation led to a ~66 % decrease of MBA-peak intensity compared to those without magnetic separation, suggesting a successful minimization of the amount of unleashed MBA/DNA_2_-Au_13_NPs in the background (Figure S22). Subsequently, we evaluated the sensitivity of this magnetic-responsive function for detecting target DNA. Strikingly, this magnetic separation caused the decrease of MBA-peak intensity to be statistically significant compared to the blank or scrambled groups after the detection of Orf-cDNA at 1 aM (as low as ~600 copies/mL; Figure S23) with a linear relationship between the difference of MBA-peak intensities and the log concentrations of the target DNA from 1 aM to 1 nM (Figure 4D-G). According to the equation of LOD calculation (the blank value plus 3 times of standard deviation) 41, LOD was estimated to be 1 aM, which is consistent with our experimental results (Figure S23; Table S3). This result implies the importance of incorporating magnetic-responsive functionality into our platform to improve the sensitivity without relying on pre-amplification of target DNA.

### Stability study of the biosensing system under various conditions

**Figure 5:**
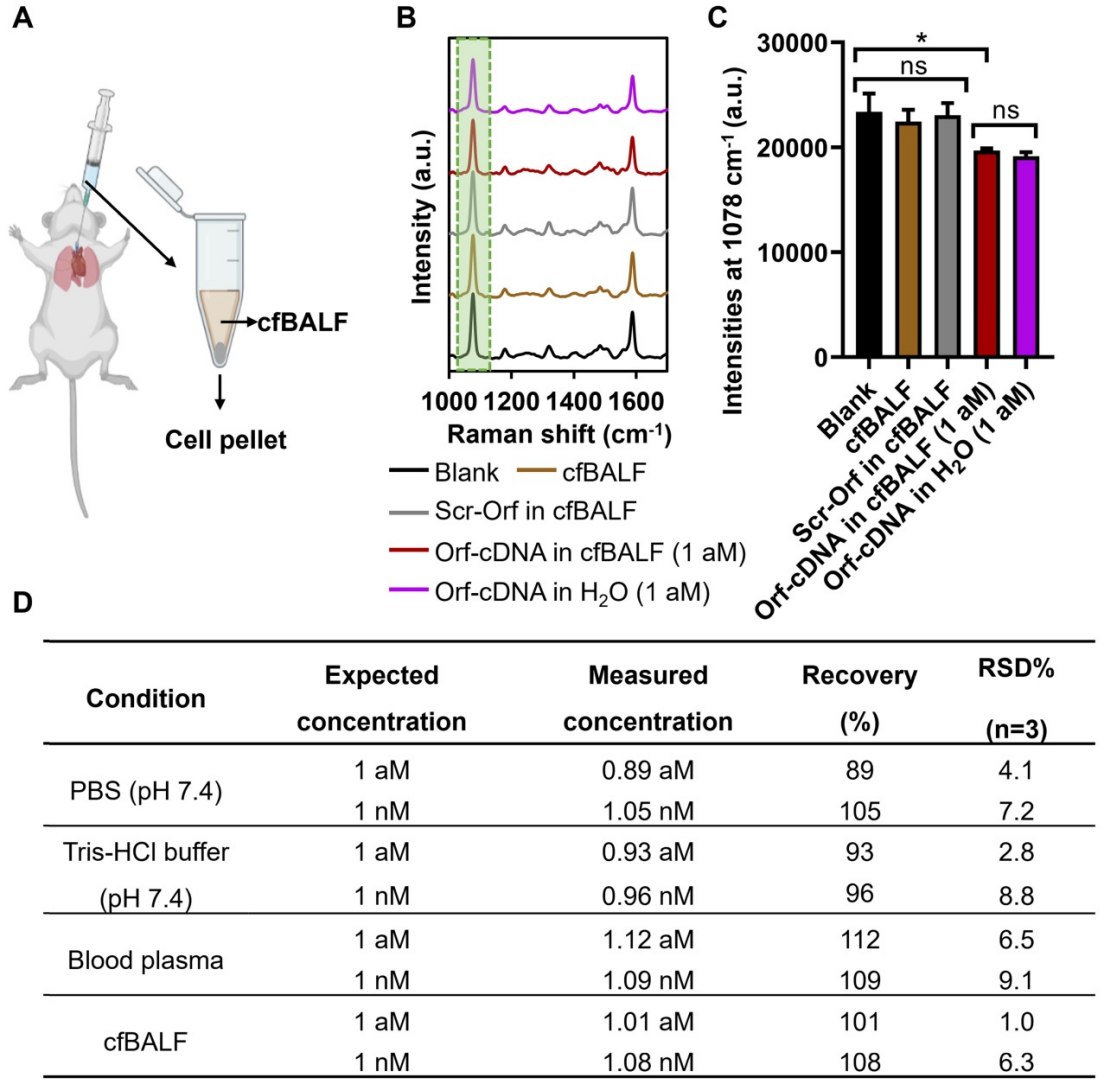
** Detection of Orf-cDNA in cell-free bronchoalveolar lavage fluid (cfBALF) using the magnetic-responsive biosensing platform.** (A) Illustration of cfBALF collected from naïve Balb/c mice. (B) SERS spectra of the biosensing platform after detecting blank samples, cfBALF, Scr-Orf in cfBALF (1 nM), Orf-cDNA in cfBALF (1 aM), and Orf-cDNA in water (H_2_O, 1 aM), displayed from bottom to top. (C) MBA-peak intensities of the biosensing platform after detecting samples, corresponding to (B). (D) Stability test of the magnetic-responsive biosensing platform on detecting Orf-cDNA under various conditions. Error bar denotes the standard deviation resulting from three independent experiments. Statistical analysis of pairwise comparison was determined by one-way ANOVA. No significance (ns): P > 0.05; * P < 0.05.

Furthermore, we assess the stability of our magnetic-responsive biosensing platform when detecting the target DNA in various buffers, murine blood plasma, and murine cell-free bronchoalveolar lavage fluid (cfBALF). BALF is a complex biological fluid that contains pulmonary immune cells, surfactant proteins, and lipids relevant to diagnosing respiratory diseases 42-44. Thus, we employed our platform to detect Orf-cDNA from a mixture of Orf-cDNA with cfBALF. Consistently, our results showed that the magnetic-responsive biosensing system could detect Orf-cDNA as low as 1 aM in cfBALF. However, the resulting signals remained similar in the presence of blank samples, Scr-Orf, and cfBALF only (Figure 5A-C). Moreover, our platform showed excellent reliability and repeatability (with the % recovery values of 89~112% and %RSD < 10%) for detecting Orf-cDNA with defined concentrations in cfFBALF, blood plasma, and various buffers (Figure 5D). These data prove that our magnetic-integrated platform can maintain high stability and reproducible signals in biological fluids such as cfBALF that present plenty of non-specific nucleic acids and proteins in the background.

**Figure 6:**
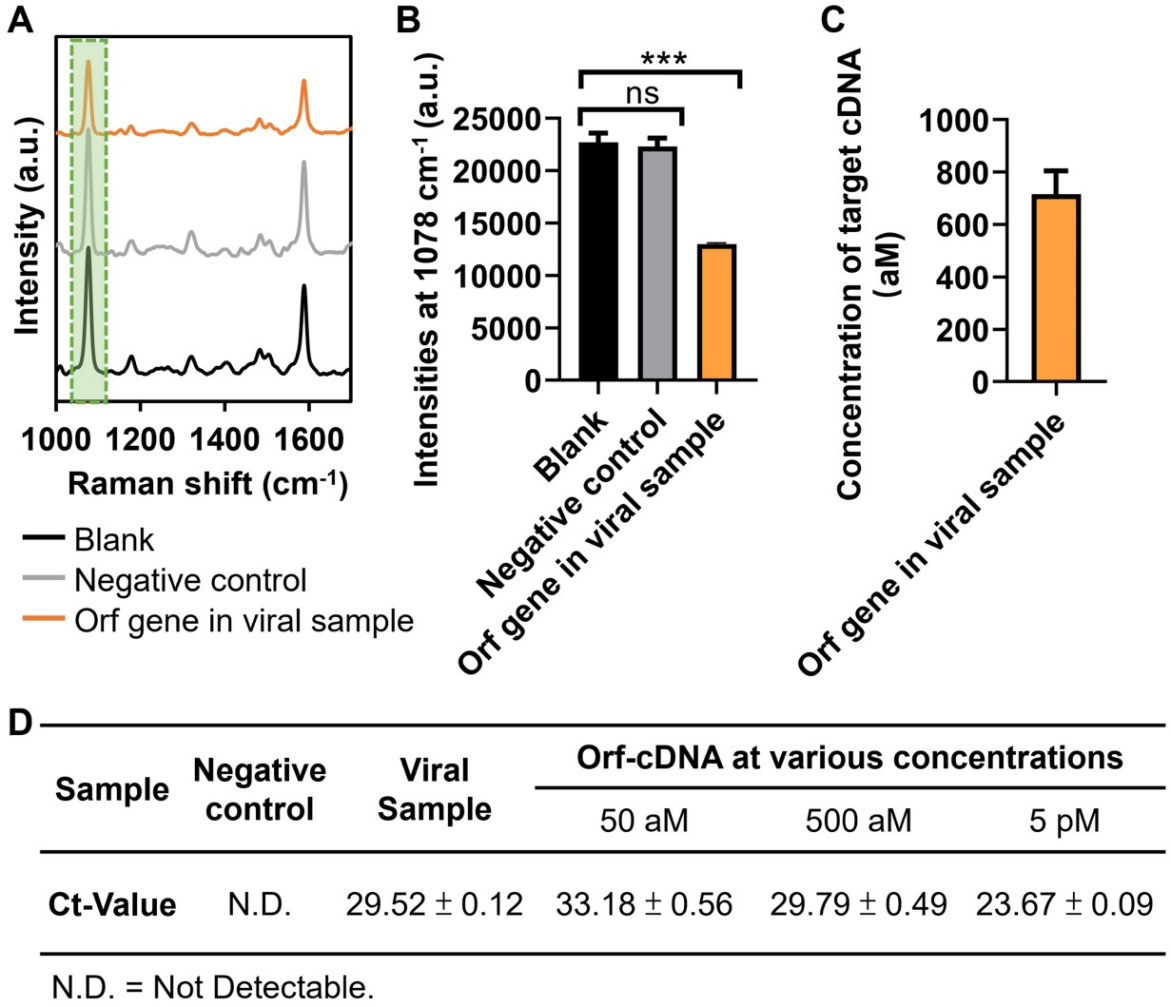
**Application of magnetic-responsive platform to detect Orf gene in cell culture-derived viral cDNA.** (A) SERS spectra of the biosensing platform after detecting blank samples, negative control, Orf sequence of interest in viral samples, displayed from bottom to top. (B) MBA-peak intensities of the biosensing platform after detecting samples, corresponding to (A). (C) Estimated concentration of Orf gene of interest in viral samples. Error bar denotes the standard deviation resulting from three independent experiments. Statistical analysis of pairwise comparison was determined by one-way ANOVA. No significance (ns): P > 0.05; *** P < 0.001. (D) RT-qPCR results of Orf sequence of interest (the same as Orf-cDNA in this study) in viral samples. Orf-cDNA groups with various concentrations serve as positive control groups, while the cell lysate group without viruses serves as a negative control. Error bar denotes the standard deviation resulting from four independent experiments.

Finally, we investigate whether our magnetic-responsive platform can detect the cDNA (reverse-transcribed RNA) of Orf gene in cell culture-derived viral samples (B.1.1.529/Omicron; GenBank: OM212472) that were obtained from our collaborative research team. The viral cDNA sample contained a large amount of non-specific DNA, especially from the cell culture, thereby being suitable to further examine the detection stability of this platform. Significantly, the presence of viral samples induced “on-off” mechanism in our platform when compared to that of the blank and negative control (cell lysates without viruses) groups (Figure 6A-B). Also, the estimated concentrations of Orf gene in the viral samples were 716 ± 89 (Figure 6C), according to the linear regression in Figure 4G. The %RSD was < 1% for detecting Orf gene from 3 independent measurements, further confirming the repeatability of detecting cell culture-derived viral cDNA samples by our platform (Figure S24) 45. In addition, we also conducted a quantitative polymerase chain reaction (qPCR), the gold standard for nucleic acid detection, to validate the detection results of our platform. First, we obtained the qPCR-calculated Ct-value of the Orf-cDNA with defined concentrations of 50 aM, 500 aM, and 5 pM to be 33.18 ± 0.56, 29.79 ± 0.49 and 23.67 ± 0.09, respectively (Figure 6D). Next, we used qPCR to measure the Ct-value of Orf gene in the viral samples. Different from the undetectable Ct-value in the cell lysate group without viruses, the Ct-value of Orf gene in the viral samples was 29.52 ± 0.12, which is similar to the Ct-value of 500 aM. Therefore, our qPCR data indicate that the Orf gene in the viral sample is detectable, and its concentration is relatively close to 500 aM in the standard Orf-cDNA samples. Together, our sensing platform provides an effective, reliable, and sensitive approach for detecting target DNA without pre-amplification.

## Conclusion

We have reported a simple, ultrasensitive, and highly selective CRISPR-Cas12a/SERS platform with a chimeric DNA/RNA hairpin guide for nucleic acid detection. Without the target amplification, our platform is optimized to detect Orf-cDNA by the internal CRISPR-Cas12a-based amplification and nanocluster-based hot spot amplification of SERS signals, which further outlines the importance of our platform design of synergistically integrating the SERS technique with CRISPR-Cas12a for ultrasensitive nucleic acid detection. Also, it shows relatively good performances among other CRISPR-based amplification-free nucleic acid biosensors, such as a low LOD, wide linear dynamic range, and high accuracy (Table S4). Conceptually, our advanced nanobiosensing platform is based on CRISPR-Cas12a/crRNA detecting target DNA to amplify the number of available RNA with the specific sequence to swift SERS intensity of the ultrabright Raman reporter from “on” to “off” as signal readouts. To the best of our knowledge, we believe that this is the first report to introduce a specifically designed displacer to resolve the steric hindrance of nanomaterials, which is hardly overcome by CRISPR-Cas12a, for hot spot removal at narrow gaps among NPs. The addition of a magnetic-responsive element in our system further improves the LOD from 10 aM (~6000 copies/mL) to 1 aM (~600 copies/mL). More importantly, this integrated platform displays excellent detection stability and repeatability and is capable of detecting the target DNA in various biological conditions. Overall, our study paves the way for tailoring the design of CRISPR-Cas12a/SERS integrated system for assisting the detection of other nucleic acid sequences.

## Materials and Methods

### Preparation of target, nontarget, mismatched, scrambled DNA sequences, and DNA strands for AuNP functionalization

The target DNA sequence was selected from a short fragment of Orf gene of SARS-CoV-2 (Orf-cDNA). The scrambled sequence (Scr-Orf) with the same length of Orf-cDNA served as a negative control. The DNA sequence of hepatitis B virus (HBV) and cDNA of N gene of SARS-CoV-2 (N-pro), human immunodeficiency virus type 1 (HIV-1), human immunodeficiency virus type 2 (HIV-2), influenza A virus (InfA), N gene of SARS-CoV-1, together with single- and double-mismatched Orf-cDNA (1MM-/2MM-Orf), were included for the selectivity study. A pair of ssDNA, DNA_1_ and DNA_2_, or DNA_1HL_ and DNA_2HL_, were used for the linkage between AuNPs with a diameter of 40 nm and 13 nm. The specifically designed chimeric DNA/RNA hairpins (1-nt, 3-nt, or 6-nt displacer) containing an ssDNA loop and a hybridized DNA/RNA were designed for the dissociation of hybridized NPs. F-displacer-D is a hairpin-based molecular beacon with the same sequence as the 6-nt displacer but is modified with a fluorophore (FAM) on the 5' of RNA_2*_ and a quencher (DABCYL) on 3' of DNA_1*_. RNA_2_ or FAM-RNA_2_, fully complementary to DNA_1_, was included as a positive control for the dissociation of hybridized NPs. crRNA for Orf-cDNA was designed to refer to the sequence of Orf-cDNA. All the nucleic acid sequences (listed in Table S1) were synthesized and purified by the Shanghai Dina Biotechnology Company. The lyophilized DNA was resuspended in DNase/RNase-free Ultrapure water (Thermo Fisher Scientific) and kept at -20 °C as a stock solution at 100 μM. The lyophilized crRNA and displacer were resuspended in diethylpyrocarbonate (DEPC)-treated water (Thermo Fisher Scientific) and kept at -80 °C as a stock solution at 100 μM.

### Preparation of citrate-capped AuNPs with a diameter of 13 nm (Au_13_NPs)

AuNPs of ~13 nm in diameter were synthesized by following Frens' method 32. After bringing 50 mL of 1 mM Au(III) chloride trihydrate (HAuCl_4_·3H_2_O, Au^3+^; Sigma-Aldrich) solution to boil, 5 mL of sodium citrate (1% w/v) (Alfa Aesar) was added under vigorous stirring, and the mixture was kept boiling for 15 min. The reaction product was slowly cooled down to room temperature (RT).

### Preparation of citrate-capped AuNP with a diameter of 40 nm (Au_40_NPs)

AuNPs of ~40 nm in diameter were prepared by an established seed-mediated growth method 46, 47. Briefly, 30 mL of 2.2 mM sodium citrate was boiled to reflux in a three-necked round-bottom flask under rapid stirring, followed by a quick injection of 0.2 mL of 25 mM Au^3+^ solution. The reaction mixture was boiled for 15 min, and a color change from colorless to reddish-orange was observed for the “seed solution”. Afterward, the reaction mixture was cooled to 90 °C and maintained at this temperature. 0.2 mL of 25 mM Au^3+^ solution was injected twice at 30 min intervals. The reaction mixture was diluted by replacing 11 mL of the solution with 10.6 mL of Nanopure water (Thermo Fisher Scientific) and 0.4 mL of 60 mM sodium citrate. At 90 °C, the addition of 0.2 mL of 25 mM Au^3+^ solution was repeated three times every 30 min. The mixture was used for subsequent NP growth. The cycle of (1) dilution, (2) injection of sodium citrate, and (3) addition of three doses of Au^3+^ solution was repeated until the resultant AuNPs measured ∼40 nm in hydrodynamic diameter.

### Loading of thiol (HS)-terminated DNA oligonucleotides to the surface of Au_13_NPs and Au_40_NPs

DNA coating procedure followed a previous report 48. DNA oligonucleotides were pre-treated by tris(2-carboxyethyl)phosphine hydrochloride (TCEP; Sigma-Aldrich) at a molar ratio of 1:200 for 1 h at RT to activate their thiol modifications. After that, the pre-treated DNA oligonucleotides were added to 1 mL of 10 nM Au_13_NPs or 0.7 nM Au_40_NPs at a molar ratio of 500:1 or 4000:1, respectively. The mixture of DNA and AuNP was manually shaken for a few seconds and tuned to 0.01% sodium dodecyl sulfate (SDS; Thermo Fisher Scientific) and 1× Tris-acetate-ethylenediaminetetraacetic acid (EDTA, TAE) buffer (Beyotime). Sodium chloride (NaCl; Sigma-Aldrich) solution was sequentially added to the NP solution at time intervals of 30 min up to a final concentration of 0.7 M to achieve dense coverage of the NP surface with DNA strands. The functionalized AuNPs were purified by four rounds of centrifugation at 12,000 × g for 15 min. Synthesized DNA_2_-Au_13_NPs and DNA_1_-Au_40_NPs were resuspended in Nanopure water at a concentration of 10 nM and 0.7 nM, respectively, and stored at 4 °C for future use.

### Loading of 4-mercaptobenzoic acid (MBA) to the vacancies of DNA_2_-Au_13_NPs (MBA/DNA_2_-Au_13_NPs)

Synthesized DNA_2_-Au_13_NPs were resuspended in Nanopure water at a concentration of 10 nM. MBA stock with a concentration of 2 mg mL^-1^ was prepared by dissolving MBA in absolute ethanol (Anaqua). MBA stock was diluted with Nanopure water to a final concentration of 1 mM and then added to 1 mL of DNA_2_-Au_13_NPs at a molar ratio of 2500:1. The mixture was shaken at RT for 1 h, followed by purification by three rounds of centrifugation at 12,000 × g for 15 min and resuspension in Nanopure water. Synthesized MBA/DNA_2_-Au_13_NPs were resuspended in Nanopure water and stored at 4 °C for future use.

### Quantification of the loading density of DNA oligonucleotides and MBA molecules

These procedures were based on a previously published report 25. DNA oligonucleotides were added to 1 mL of 10 nM Au_13_NPs or 0.7 nM Au_40_NPs at a molar ratio of 500:1 or 4000:1, respectively. After centrifuging the reacted solution of DNA-AuNPs, the supernatant that contains the unbound DNA strands was collected to quantify the loading of DNA strands onto the NPs. The concentration of unbound DNA strands was determined by NanoDrop (Thermo Fisher Scientific). The consumed amount of DNA strands was calculated by subtracting the number of unbound DNA strands from the original amount of DNA strands that were added to the AuNPs. The density of MBA molecules on the surface of DNA_2_-Au_13_NPs was determined by thiol depletion *via* the Ellman's assay. 1 mL of 10 nM DNA_2_-Au_13_NPs was mixed with 1 mM MBA a molar ratio of 2500:1 for 1 h under stirring. After centrifuging the NP solution at 12000 × g for 15 min, the supernatant that contains the unreacted MBA molecules was collected. 50 µL of the resultant MBA sample was mixed with 20 μL of Ellman's assay buffer [1 mM EDTA (Sigma-Aldrich) in 0.1 mM Na_2_HPO_4_ (Sigma-Aldrich); pH = 8] and 50 μL of Ellman's detection buffer [0.5 mg/mL of Ellman's reagent (5,5-dithio-bis(2-nitrobenzoic acid)) (JenKem Technology) in the assay buffer]. DNA_2_-Au_13_NPs not functionalized with MBA molecules were included as a negative control. After 30 min of continuous shaking, the absorbance of the reaction mixture was read at 412 nm by a Varioskan LUX Multimode microplate reader (Thermo Fisher Scientific). The concentration of MBA was calculated with reference to a standard calibration curve after subtracting the background absorbance of the sample derived from that of the negative control. Reported data represent mean ± SD from three independent experiments.

### Hybridization and dissociation of DNA_1_-Au_40_NPs and MBA/DNA_2_-Au_13_NPs by incubation at a temperature higher than its “melting” temperature (Tm)

DNA_1_-Au_40_NPs and MBA/DNA_2_-Au_13_NPs were mixed at a molar ratio of 1:2.5, 1:5, 1:7.5, and 1:10 in a hybridization buffer [0.1 M NaCl, 10 mM phosphate buffer [NaH_2_PO_4_ (Sigma-Aldrich) and Na_2_HPO_4_, pH 7.0], and incubated at RT overnight. For the hybridization of DNA_1HL_-Au_40_NPs and MBA/DNA_2HL_-Au_13_NPs were mixed at a molar ratio of 1:5 and incubated at RT overnight. For the dissociation of hybridized DNA_1_-Au_40_NPs and MBA/DNA_2_-Au_13_NPs (at the optimal ratio of 1:5), the hybridized NPs were concentrated by centrifugation at 8,000 × g for 5 min and resuspended in Nanopure water, followed by incubation at 65 °C for 30 min.

### Trans-cleavage of the DNA loop in the displacer by CRISPR-Cas12a in the presence of target DNA sequence

Lachnospiraceae bacterium ND2006 Cas12a (LbCas12a) reaction kit was purchased from Magigen Biotech company. The CRISPR-Cas12a-mediated cleavage assay was conducted referring to the user instructions and previous reports 49, 50. Firstly, LbCas12a (1 μL, 2 μM), crRNA (2 μL, 5 μM) were mixed to form LbCas12a/crRNA complex and target DNA (1 μL) with various concentrations or nontarget DNA sequences (1 μL, 10 nM) or cleavage buffer (blank, 1 μL) and 1-nt, 3-nt, 6-nt displacer or F-displacer-D (2.5 μL, 10 μM) were added to form a 10-μL reaction solution. The cleavage reaction was performed at 37 °C for 0.5 h. Note that the F-displacer-D is a hairpin-based molecular beacon with the same sequence as the 6-nt displacer in our detection system but is modified with a fluorophore (FAM) on the 5' of RNA_2*_ and a quencher (DABCYL) on 3' of DNA_1*_ (Table S1). Resultant DNA was run in a 4 % agarose gel stained by Sybr (1:10000; Thermofisher) in 0.5× TBE buffer [Tris (Sigma-Aldrich), 44.5 mM; boric acid (Sigma-Aldrich), 44.5 mM; EDTA, 1 mM; pH 8.0] with a voltage of 80 V to confirm the successful cleavage of the displacer. F-displacer-D treated by target-activated CRISPR-Cas12a and FAM-RNA_2_ were measured by fluorescence spectroscopy (Agilent Cary Eclipse).

### Dissociation of hybridized DNA_1_-Au_40_NPs and MBA/DNA_2_-Au_13_NPs by CRISPR-Cas12a-treated displacers

RNA_2_ (10 μL) with the same concentration as that of the displacers was added to hybridized DNA_1_-Au_40_NPs and MBA/DNA_2_-Au_13_NPs (40 μL) in a hybridization buffer. To optimize the duration of the displacement, the reaction was performed at 37 °C for 0.5, 1, 2, 3 h under gently rotating. Collaterally cleaved displacer (10 μL) was added to hybridized DNA_1_-Au_40_NPs and MBA/DNA_2_-Au_13_NPs (40 μL) in a hybridization buffer. The displacement was performed at 37 °C for 2 h under gentle rotation.

### Preparation of magnetic AuNPs with superparamagnetic iron oxide core (Fe@Au_40_ NPs)

These procedures were based on a previously published report 51. Firstly, a superparamagnetic FeNP core was synthesized. In a typical procedure, 175 mg of iron sulfate (FeSO_4_, 1.152 µmol; Aladdin), 2.5 mL of 2.0 M sodium nitrate (NaNO_3_; Sigma-Aldrich), 2.5 mL of 1.0 M sodium hydroxide (NaOH; International laboratory USA), and 5 mL of 8 mg/mL polyethyleneimine (PEI, branched, M_W_ ∼25000; Aladdin) were added to 20 mL of nitrogen-purged ultrapure water. Under the oxygen-free environment, the mixture was heated to 90 °C under vigorous stirring, with continued heating at this degree for 2 h. During the heating process, the solution changed color from blue to black, indicating the formation of FeNPs. The NPs were purified by magnetic separation for five cycles and then redispersed in 20 mL of ultrapure water for further use. Secondly, we attached Au seeds to FeNPs to facilitate the growth of the Au shell. 5 nm AuNPs were synthesized according to the previous report 52. In a typical procedure, 55 μL of 0.32 mg of PEI-stabilized FeNPs was added to 5 mL of as-prepared 5 nm AuNPs and stirred for 45 min. After purification by three cycles of magnetic separation and washing, the solution was redispersed in 2.5 mL of 15 mg/mL PEI aqueous solution and heated at 60 °C for 3 h. After purifications by three cycles of magnetic separation and washing, the Fe-Au NPs were redispersed in 1 mL of Nanopure water for further use. Lastly, to synthesize Fe@Au_40_ NPs, an Au growth solution was prepared by adding 200 μL of 10 mM HAuCl_4_ into 5 mL of 0.1 M cetyltrimethylammonium bromide (CTAB; Tokyo Chemical Industry Co., Ltd.) aqueous solution. After mixing for 5 min, 32 μL of 100 mM ascorbic acid (AA; Sigma-Aldrich) was added to reduce HAuCl_4_ to HAuCl_2_ and stirred for 30 s. Then, 300 μL of pre-synthesized Fe-Au NPs were injected. The solution was stirred for 30 s followed by incubation for 2 h to allow complete growth of Fe@Au_40_ NPs. The synthesized Fe@Au_40_ NPs are ~40 nm further modified and characterized following similar procedures to that of Au_40_NPs.

### Characterization of various types of NPs

The ultraviolet-visible (UV-vis) spectra of AuNPs, modified AuNPs, Fe@Au_40_ NPs, and modified Fe@Au_40_ NPs were obtained by UV-vis spectrophotometer (Ultrospec 2100 pro). The concentration of NPs solution was determined by their UV-vis spectra based on Beer-Lambert's law and the molar extinction coefficient of AuNPs of 13 nm or 40 nm in diameter at 450 nm (1.39 × 10^8^ M^-1^ cm^-1^ or 4.92 × 10^9^ M^-1^ cm^-1^, respectively) 53. Hydrodynamic diameters and ζ-potentials were measured by the Zetasizer Nanosystem (Malvern Instruments). Reported values represent mean ± SD from three independent measurements. The NPs were visualized by transmission electron microscope (TEM) at a voltage of 100 kV (Hitachi H7700). To confirm the DNA coating on the surface of NPs, NPs were negatively stained for 5 min using platinum blue ([Pt_4_(NH_3_)_8_(C_6_H_13_O_5_)_4_]) from Nisshin EM Co., Ltd. (Tokyo, Japan). The magnetic properties of the Fe@Au_40_ NPs were characterized by a vibrating sample magnetometer (VSM; Lake Shore 7400 Series) operating with ramping rate and data point 80 Oe/step at RT. Powder X-ray diffraction (PXRD) patterns of FeNPs and Fe@Au_40_ NPs were collected at 293 K with a Rigaku SmartLab X-Ray diffractometer using Cu Kα radiation. Elemental mapping was performed using a scanning transmission electron microscopy (STEM; Tecnai F20, FEI) equipped with an EDX analysis system (Oxford Instruments). NP imaging was carried out in HAADF-STEM mode at a beam voltage of 200 kV.

### Dissociation of DNA_1_-Fe@Au_40_ NPs and MBA/DNA_2_-Au_13_NPs by magnetic separation

Similar to DNA_1_-Au_40_NPs, DNA_1_-Fe@Au_40_ NPs were hybridized with MBA/DNA_2_-Au_13_NPs at a molar ratio of 1:5 and incubated at RT overnight. The hybridized NPs (40 μL) were treated using a cleaved displacer (10 μL) in a hybridization buffer at 37 ◦C for 2 h under gentle rotation. The dissociated NPs were placed close to a permanent magnet (35 mm × 10 mm × 3 mm; Hong Kong WAHFA Magnet company) for 10 min to separate the magnetic DNA_1_-Fe@Au_40_ NPs from MBA/DNA_2_-Au_13_NPs. The magnetic strength of the permanent magnet was estimated by 410 Hand-held Gaussmeter (Lake Shore Cryotronics) along with various distances from the Gaussmeter. The supernatant without magnetic property was discarded, and the magnetic pellet was resuspended in a hybridization buffer for Raman measurement.

### Raman signal measurement of this biosensing platform

2 μL of the liquid sample was dropped on the silicon wafer that was pretreated by (3-aminopropyl)triethoxysilane (APTES) according to our previous report 25, with gentle shaking at 200 rpm and 37 °C, to evaporate and deposit on the wafer via electrostatic interaction within several minutes. Within the center area of the droplet (the diameter < 200 µm), 6 random points were measured by Renishaw Micro-Raman Spectroscopy with a 50 × working objective lens. Due to the gentle shaking and electrostatic interaction between the DNA-coated AuNPs (negatively charged) and the APTES-treated silicon wafer (positively charged), the aggregation can be largely avoided during the process of sample preparation. The sample was excited by a 785 nm laser with 5% laser power and 10 s exposure time. All of the obtained Raman spectra were zoomed into regions to reveal Raman bands with baseline correction with the graphic plot by Origin 2018 software.

### Collection of bronchoalveolar lavage fluid (BALF) and blood from mice

The Animal Subjects Ethics Sub-committee (ASERSC) of the Hong Kong Polytechnic University approved all the experiments listed below (20-21/166-BME-R-GDG). Bronchoalveolar lavage fluid (BALF) was collected by lavaging the lung tissue of naïve Balb/c mice three times with 0.5 mL of phosphate-buffered saline (PBS) following a previous report 44. Cell-free BALF (cfBALF) were collected by centrifugation at 1800 rpm for 10 min at 4 oC and discarding the cell pellet. Blood was collected according to a previous report 54. A total of 0.5 mL of blood was drawn by cardiac puncture and stored inside EDTA-coated tubes (Becton Dickinson). After centrifugation at 1500g for 10 min, 0.2 mL of the blood plasma in the supernatant was collected.

### Stability study of the biosensing system for detecting Orf-cDNA under various conditions

Orf-cDNA with defined concentration was suspended in PBS (pH 7.4), Tris-Hydrochloride (HCl) buffer (20 mM, pH 7.4), cfBALF, and blood plasma. Orf-cDNA in different buffers with defined concentrations was loaded to the CRISPR-Cas12a reaction solution for detection.

### Virus and cell line

All experiments involving live B.1.1.529/Omicron (GenBank: OM212472) followed the approved standard operating procedures of the Biosafety Level 3 facility at The University of Hong Kong. Vero-E6-TMPRSS2 cells were maintained in Dulbecco's Modified Eagle's Medium (DMEM; Gibco) containing 10% fetal bovine serum (FBS; Gibco), 2 mM L-glutamine (Thermo Fisher Scientific), 100 U/mL/mL penicillin (Thermo Fisher Scientific), and incubated at 37 oC in a 5% CO_2_ setting. After obtaining the virus culture, the sequence of B.1.1.529/Omicron used in this study was confirmed with nanopore sequencing.

### Detection of Orf-cDNA in viral samples by quantitative reverse transcription polymerase chain reaction (RT-qPCR)

We performed RT-qPCR according to a previous report 55. Basically, RNAiso plus (Takara) was used to extract total RNA, including viral RNA, in cultured cells according to the manufacturer's instructions. The concentrations of extracted RNA were measured by a NanoDrop. Then, the extracted RNA was reverse transcripted into cDNA using a PrimeScript™ RT reagent Kit (Perfect Real Time) according to the manufacturer's instructions (Takara). After that, we carried out RT-qPCR using Taqman primers probing the Orf1ab gene using CFX96 Real-Time System (Bio-Rad Laboratories). Cells without viruses were included as the negative control. Sequences of primers are listed in Table S1.

### Finite-difference time-domain (FDTD) simulation

The optical simulation was conducted by Lumerical FDTD Solution. The radius of AuNPs was set as 6.5 nm and 20 nm, respectively, referring to TEM measurement. The distance between the two AuNPs was estimated to be 10 nm, according to the length of hybridized (double-stranded DNA; dsDNA) and unhybridized (ssDNA) domains of DNA_1_ and DNA_2_ 36. The MBA molecules adsorbed on MBA/DNA_2_-Au_13_NP were set to be within 1.5 nm of the AuNP surface. The dielectric data of the Au_13_NP-Au_40_NP model were adopted from empirical data given by Johnson and Christy 56. Total field-scattered field (TSFS) was used as an excitation light source, and the K vector was set perpendicular to the vertical interparticle separation. The whole simulation area was bounded by a perfect match layer (PML) 57. For simplicity, symmetric and anti-symmetric boundary conditions were applied to reduce the simulation time.

### Statistical analysis

For each experiment, we employed three silicon wafers for detection (n = 3). The Raman spectra were averaged from three independent samples. For each silicon wafer sample, we measured 6 random points. The statistical analysis of pairwise comparison was obtained by one-way ANOVA.

## Supplementary Material

Supplementary figures and tables.

## Abbreviations

AuNPs: gold nanoparticles
Au_40_NPs: 40 nm AuNPs
Au_13_NP: 13 nm AuNPs
FeNPs: superparamagnetic iron oxide nanoparticles
SARS-CoV-2: severe acute respiratory syndrome coronavirus 2
FET: field effect transitor
CRISPR-Cas12a: clustered regularly interspaced short palindromic repeats-associated nuclease 12a
crRNA: CRISPR RNA
ssDNA: single-stranded nucleic acids
SERS: Surface-enhanced Raman scattering
EMF: electromagnetic fields
LSPR: localized surface plasmon resonance
SPR: surface plasmon resonance
TMSDR: toehold-mediated strand displacement reaction
FRET: Förster resonance energy transfer
MBA: 4-mercaptobenzoic acid
LOD: limit of detection
%RSD: percent relative standard deviation
TEM: transmission electron microscope
UV-vis: ultraviolet-visible
DLS: dynamic light scattering
FDTD: finite-difference time-domain
1MM: single-mismatched
2MM: double-mismatched
Orf: open reading frames 1ab
qPCR: quantitative polymerase chain reaction
Ct: cycle threshold
HBV: hepatitis B virus
HIV-1: human immunodeficiency virus type 1
HIV-2: human immunodeficiency virus type 2
InfA: influenza A
N-pro: nucleocapsid protein
FAM: carboxyfluorescein
DABCYL: 4-((4-(dimethylamino)phenyl)azo)benzoic acid
F-displacer-D: DNA/RNA chimeric hairpin-based molecular beacon
cfBALF: cell-free bronchoalveolar lavage fluid
