# Supplementary material for: A CRISPR-Cas12a integrated SERS nanoplatform with chimeric DNA/RNA hairpin guide for ultrasensitive nucleic acid detection
Source: Theranostics. 2022 Aug 8;12(13):5914–30. doi: 10.7150/thno.75816 (PMC9373821; doi:10.7150/thno.75816)
Supplement: Supplementary file 1 — Supplementary figures and tables. [file thnov12p5914s1.pdf]

Supporting Information

A CRISPR-Cas12a integrated SERS nanoplatform with chimeric DNA/RNA hairpin guide for ultrasensitive nucleic acid detection

Bohan Yin[1], Qin Zhang[1], Xinyue Xia[2], Chuanqi Li[1], Willis Kwun Hei Ho[1], Jiaxiang Yan[1], Yingying Huang[1], Honglian Wu[1], Pui Wang[3], Changqing Yi[4], Jianhua Hao[5], Jianfang Wang[2], Honglin Chen[3], Siu Hong Dexter Wong[1,6,]*, Mo Yang[1,]*

1. Department of Biomedical Engineering, The Hong Kong Polytechnic University, Kowloon, Hong Kong 999077, China
2. Department of Physics, The Chinese University of Hong Kong, Shatin, Hong Kong 999077, China
3. Department of Microbiology, The University of Hong Kong, Pokfulam, Hong Kong 999077, China
4. Key Laboratory of Sensing Technology and Biomedical Instruments (Guangdong Province), School of Biomedical Engineering, Sun Yat-Sen University, Guangzhou, 510006, P. R. China
5. Department of Applied Physics, The Hong Kong Polytechnic University, Kowloon, Hong Kong 999077, China
6. Research Institute for Sports Science and Technology, The Hong Kong Polytechnic University, Kowloon, Hong Kong 999077, China

***Corresponding authors:**
Siu Hong Dexter Wong: shongwong@polyu.edu.hk
Mo Yang: mo.yang@polyu.edu.hk

Keywords: gold nanoparticles, magnetic manipulation, surface-enhanced Raman spectroscopy, CRISPR-Cas12a, nucleic acid detection

Table S1. List of oligonucleotide sequences.

Name	Sequence Profile (5' to 3')
DNA_1	AAAAAAAAATAGCTTATCAGCGCGCGAA-$(CH_2)_6$-SH
DNA_2	CTGATAAGCTATTTTTTTTTT-$(CH_2)_6$-SH
DNA_{1HL}	AAATATCAGCGCAA-$(CH_2)_6$-SH
DNA_{2HL}	CTGATATTTTT-$(CH_2)_6$-SH
RNA_2	cgcgcgcugauaagcuauuuuuuuuu
Scr-RNA_2	guucguucucguuauauagucucuag
6-nt displacer	cgcgcgcugauaagcuauuuuuuuuuATTATTATTATTATTATTATTATT ATTAAAAAAAAATAGCTTATCAGCGCGCG
3-nt displacer	gcgcugauaagcuauuuuuuuuuATTATTATTATTATTATTATTATTAT TAAAAAAAAATAGCTTATCAGCGC
1-nt displacer	gcugauaagcuauuuuuuuuuATTATTATTATTATTATTATTATTATT AAAAAAAAATAGCTTATCAGC
F-displacer-D	FAM-cgcgcgcugauaagcuauuuuuuuuuATTATTATTATTATTATTATTATT ATTAAAAAAAAATAGCTTATCAGCGCGCG-DABCYL
FAM-RNA_2	FAM-cgcgcgcugauaagcuauuuuuuuuu
Orf-crRNA	uaauuucuacuaaguguagauacgauugugcaucagcuga
Orf-cDNA	CCCTGTGGGTTTTACACTTAAAAACACAGTCTGTACCGTCTG CGGTATGTGGAAAGGTTATGGCTGTAGTTGTGATCAACTCC GCGAACCCATGCTTCAGTCAGCTGATGCACAATCGT
Scr-Orf	ATAGTAGCTCGTCCTCGAGGTGGCGTATTCGGAAGATCGTC AATCTGTATTGACTGCTCCAACATTCCAAGACGTCCTAACA CTGTGTGCTTGAGGATTCCGCGGAACAATCTTGGTAT
Orf-1MM	CCCTGTGGGTTTTACACTTAAAAACACAGTCTGTACCGTCTG CGGTATGTGGAAAGGTTATGGCTGTAGTTGTGATCAACTCC GCGAACCCATGCTTCAGTCAGCTGATCCACAATCGT
Orf-2MM	CCCTGTGGGTTTTACACTTAAAAACACAGTCTGTACCGTCTG CGGTATGTGGAAAGGTTATGGCTGTAGTTGTGATCAACTCC GCGAACCCATGCTTCAGTCAGCTGAACCACAATCGT
N-Pro	GGGGAACTTCTCCTGCTAGAATGGCTGGCAATGGCGGTGA
HIV-1-cDNA	GCTATACATTCTTACTATTTTATTTAATCCCAG

HIV-2-cDNA	TGAATTTAGTTGCGCCTGGTCCTTT
HBV	CTGGATCCTGCGCGGGACGTCCTT
InfA-cDNA	CGTGCCCAGTGAGCGAGGACTGCA
SARS-CoV-1	AACCAGAATGGAGGACGCAATGGGGCAAGGCCAAAACAGCGCCGACCCCAAGGTTTACCCAATAATACTGCGTCTTGGTTCACAGC
Orf-f primer	CCCTGTGGGTTTTACACTTAA
Orf-r primer	ACGATTGTGCATCAGCTGA

*note that nucleotide(s) in red color indicate mutation from the target DNA sequences. The sequence in lowercase letters indicates the RNA sequence.

Table S2. Hydrodynamic diameters of DNA_1–$Au_{40}NPs$ and MBA/DNA_2–$Au_{13}NPs$ after mixture, hybridization, and dissociation.

Sample	Mixture	Hybridization	Dissociation
Hydrodynamic diameter in water at RT (nm)	49.69 ± 0.78 (0.19)	98.31 ± 2.86 (0.21)	59.71 ± 1.01 (0.15)

Numbers in parentheses refer to the polydispersity index (PDI). RT = room temperature. Error bar denotes the standard deviation resulting from three independent experiments.

Table S3. Estimated limit of detection (LOD) based on linear regression formula.

Sample	Proposed biosensing platform			
	Nonmagnetic-responsive		Magnetic-responsive	
	Estimated LOD*	Experimental LOD	Estimated LOD*	Experimental LOD
Orf-cDNA	5.27 aM[#]	10 aM	1.04 aM	1 aM

* Estimated LOD was obtained by the blank value plus 3 times of standard deviation.
[#] Note that the nonmagnetic-responsive platform was unable to detect estimated LOD experimentally.

Table S4. Current CRISPR-based amplification-free biosensors for nucleic acid detection.

Signal output	CRISPR enzyme	Target	Limit of detection	linear dynamic range	Recovery	Reference
Fluorescence	Cas12a	BRCA-1	0.34 fM	1 fM–100 pM	N.M.	[1]
Fluorescence	Cas13a	miR-17	4.5 aM	5 aM–100 fM	N.M.	[2]
Fluorescence	Cas12a	HPV-16	10 fM	N.M.	89%–99%	[3]
Electrochemical signal	Cas12a	HPV-16	50 pM	N.M.	N.M.	[4]
Electrochemical signal	Cas13a	miR-19b	10 pM	N.M.	N.M.	[5]
Raman	Cas12a	SARS-CoV-2 N gene	1 fM	N.M.	N.M.	[6]
Raman	Cas12a	SARS-CoV-2 Orf gene	1 aM	1 aM–1 nM	89%–112%	This work

N.M. = not mentioned

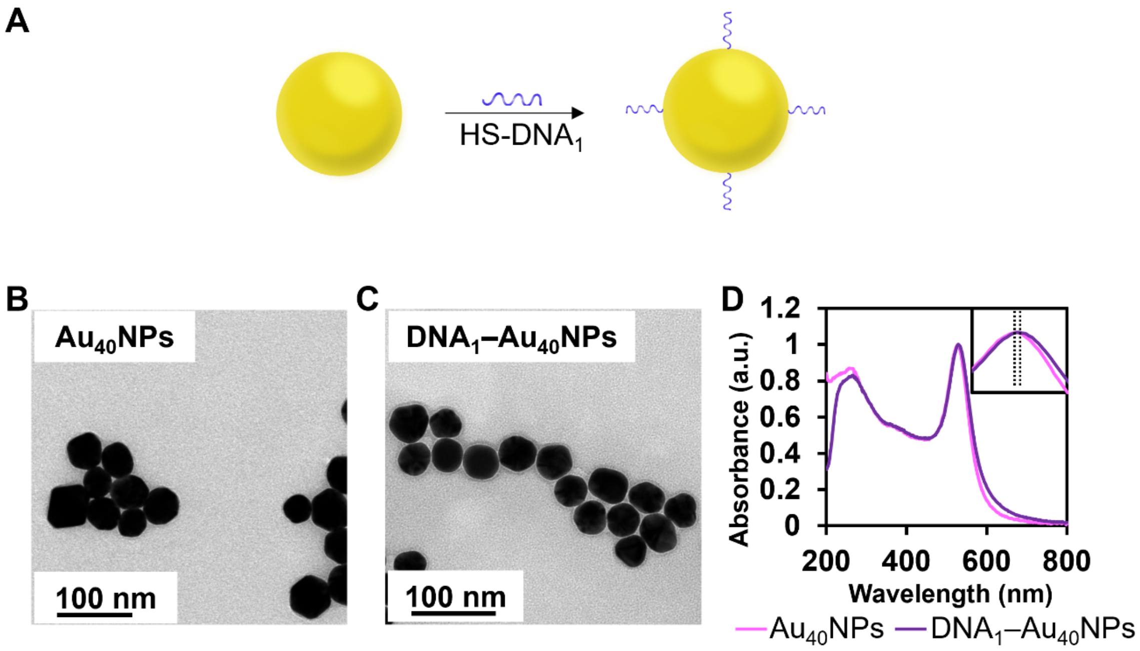

E

Sample	$Au_{40}NPs$	DNA_1–$Au_{40}NPs$
Hydrodynamic diameter in water at RT (nm)	40.23 ± 0.17 (0.08)	56.92 ± 0.51 (0.19)
ζ-potential in water at RT (mV)	-29.10 ± 1.90	-31.93 ± 0.67
No. of DNA strands/NP	N.A.	1224 ± 95

Figure S1. Preparation and characterization of DNA_1-coated $Au_{40}NPs$ (DNA_1–$Au_{40}NPs$). (A) Schematic illustration of conjugating DNA_1 primer to the surface of $Au_{40}NPs$. Representative transmission electron microscope (TEM) images of (B) $Au_{40}NPs$ and (C) DNA_1–$Au_{40}NPs$. The DNA shell surrounding the NP core was stained with platinum blue. (D) Ultraviolet-visible (UV-vis) spectra of $Au_{40}NPs$ and DNA_1–$Au_{40}NPs$ suspended in water (peak at 528 nm and 530 nm, respectively). The anhydrous diameter of $Au_{40}NPs$ was determined to be 40.79 ± 3.82 nm by measuring at least 50 NPs. (E) By dynamic light scattering (DLS) analysis, $Au_{40}NPs$ and DNA_1–$Au_{40}NPs$ had hydrodynamic diameters of 40.23 ± 0.17 and 56.92 ± 0.51 nm, respectively. Their zeta potentials were around -30 mV. By NanoDrop, the loading of DNA_1 primers on the $Au_{40}NP$ core was determined to be 1,224 ± 95 strands/NP. Numbers in parentheses refer to the polydispersity index (PDI). RT = room temperature. N.A. = not applicable. Error bar denotes the standard deviation resulting from three independent experiments.

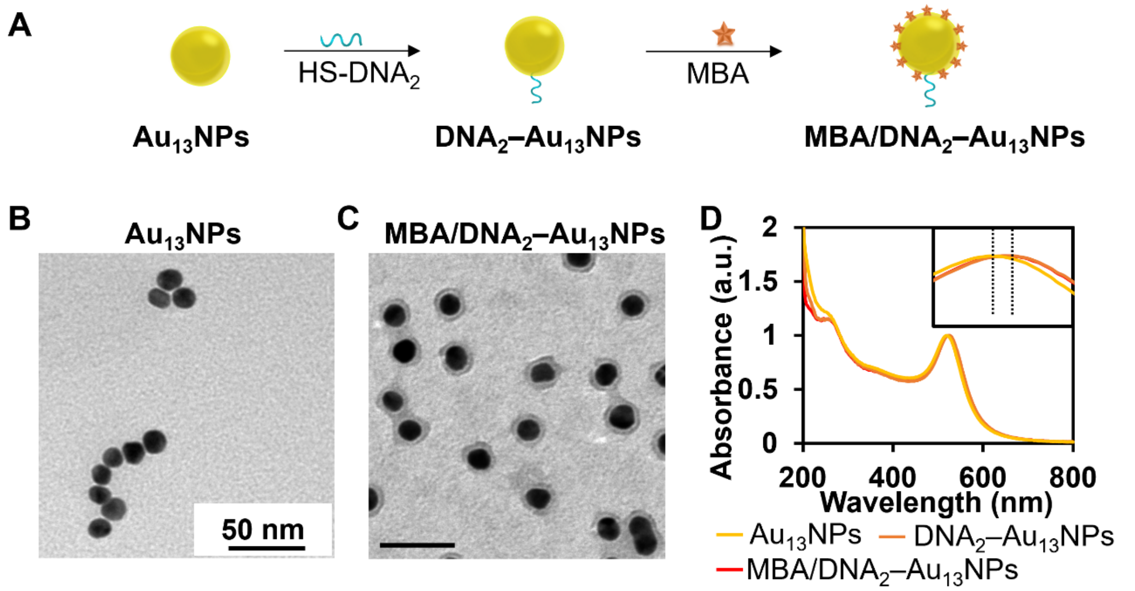

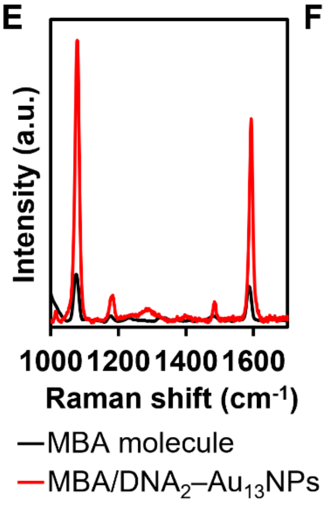

F

Sample	$Au_{13}NPs$	DNA_2–$Au_{13}NPs$	MBA/DNA_2–$Au_{13}NPs$
Hydrodynamic diameter in water at RT (nm)	19.37 ± 0.20 (0.25)	35.11 ± 1.31 (0.24)	34.14 ± 3.78 (0.29)
ζ-potential in water at RT (mV)	-32.73 ± 0.95	-33.27 ± 1.62	-35.60 ± 0.69
No. of DNA strands/NP	N.A.	149 ± 15	
No. of MBA molecules/NP	N.A.	N.A.	2344 ± 38

Figure S2. Preparation and characterization of MBA- and DNA_2-coated $Au_{13}NPs$ (MBA/DNA_2–$Au_{13}NPs$). (A) Schematic illustration of conjugating DNA_2 primer to the surface of $Au_{13}NPs$. TEM images of (B) $Au_{13}NPs$ and (C) MBA/DNA_2–$Au_{13}NPs$. The DNA shell surrounding the NP core was stained with platinum blue. The anhydrous diameter of $Au_{13}NPs$ was determined to be 13.08 ± 1.10 nm by measuring at least 50 NPs. (D) UV-vis spectra of $Au_{13}NPs$, DNA_2–$Au_{13}NPs$, and MBA/DNA_2–$Au_{13}NPs$ suspended in water (peak at 518, 522, and 522 nm, respectively). (E) The surface-enhanced Raman scattering (SERS) spectra of MBA molecule and MBA/DNA_2–$Au_{13}NPs$. (F) By DLS analysis, $Au_{13}NPs$, DNA_2–$Au_{13}NPs$, MBA/DNA_2–$Au_{13}NPs$ had hydrodynamic diameters of 19.37 ± 0.20, 35.11 ± 1.31, and 34.14 ± 3.78 nm, respectively. Their zeta potentials lay between -32 mV and -36 mV. By NanoDrop, the loading of DNA_2 primers on the $Au_{13}NP$ core was determined to be 149 ± 15 strands/NP. By Ellman's assay, the loading of MBA molecules on DNA_2–$Au_{13}NPs$ was determined to be 2,344 ± 38 molecules/NP. Numbers in parentheses refer to the PDI. RT = room temperature. N.A. = not applicable. Error bar denotes the standard deviation resulting from three independent experiments.

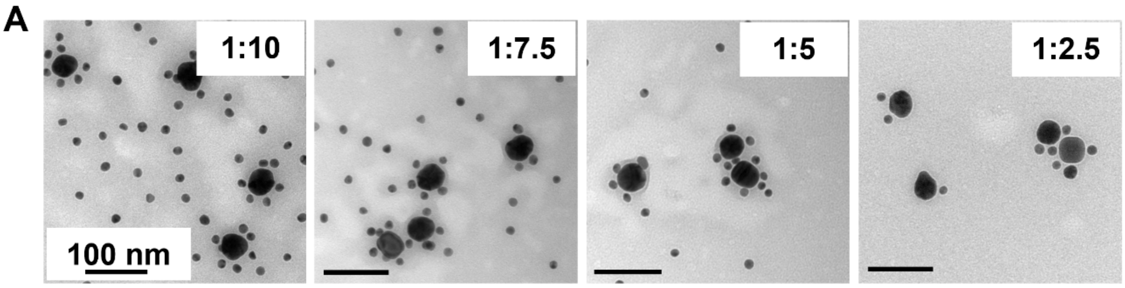

B

Ratio of DNA_1–Au_{40}NPs to MBA/DNA_2–Au_{13}NPs	1:10	1:7.5	1:5	1:2.5
No. of MBA/DNA_2–Au_{13}NPs per DNA_1–Au_{40}NP	4.64 ± 0.52	4.79 ± 0.89	4.51 ± 0.63	2.10 ± 0.20

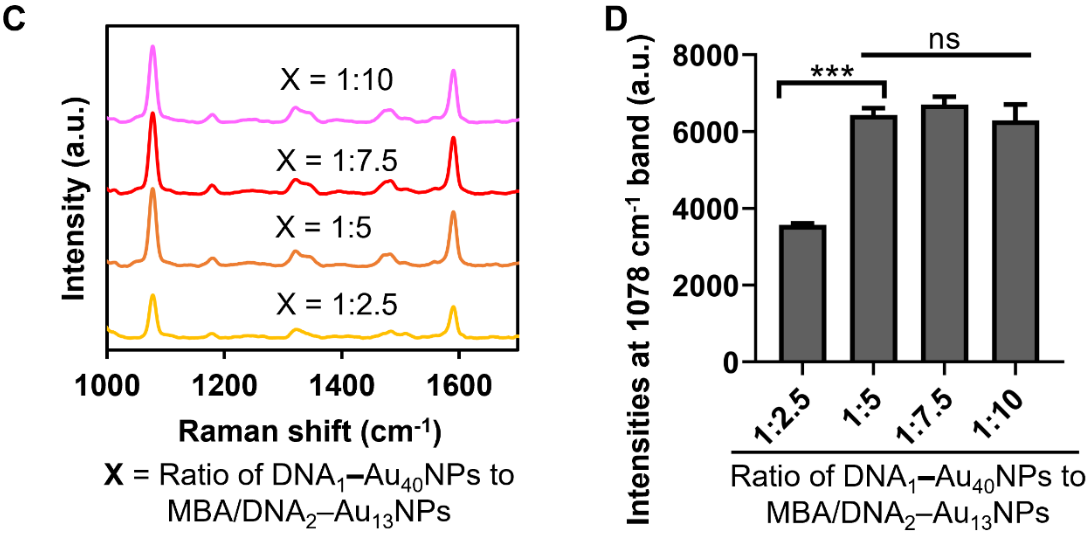

Figure S3. Comparison of Raman signals of hybridized DNA_1–Au_{40}NPs and MBA/DNA_2–Au_{13}NPs at different molar ratios. (A) Representative TEM images of hybridized NPs at different ratios of DNA_1–Au_{40}NPs to MBA/DNA_2–Au_{13}NPs. (B) Estimated number of surrounding MBA/DNA_2–Au_{13}NPs per DNA_1–Au_{40}NPs, by counting at least 50 DNA_1–Au_{40}NP cores in TEM images. (C) SERS spectra of hybridized NPs at different ratios of DNA_1–Au_{40}NPs to MBA/DNA_2–Au_{13}NPs. (D) Quantitative analysis of MBA-peak intensities (at 1078 cm^{-1}) of samples, corresponding to (C). Error bar denotes the standard deviation resulting from three independent experiments. Statistical analysis of pairwise comparison was determined by one-way ANOVA. No significance (ns): $P > 0.05$ and *** $P < 0.001$.

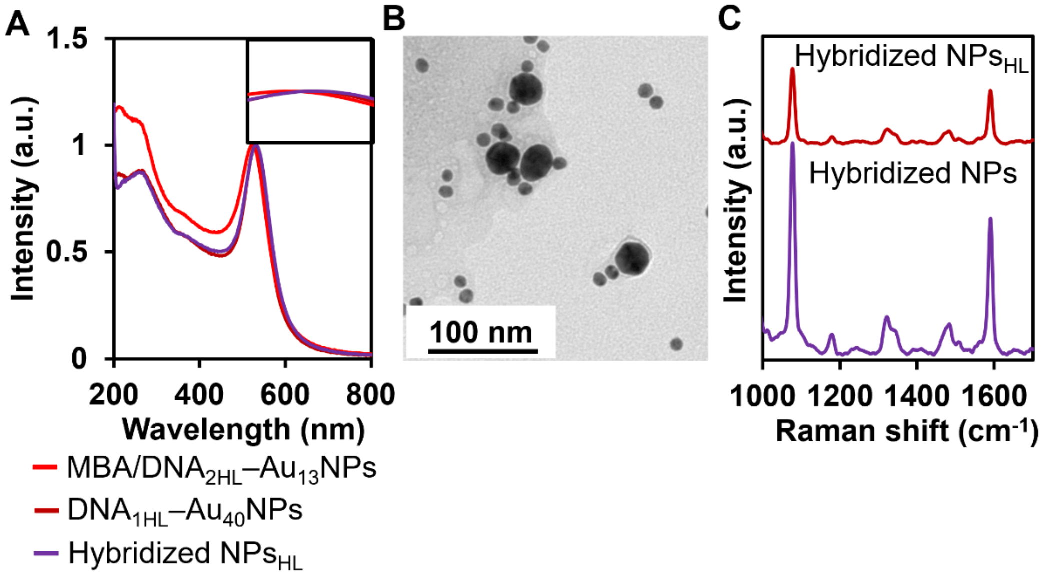

D

Sample	MBA/DNA$_{2HL}$–Au$_{13}$NPs	DNA$_{1HL}$–Au$_{40}$NPs
Hydrodynamic diameter in water at RT (nm)	25.28 ± 2.65 (0.23)	52.84 ± 3.51 (0.27)
ζ-potential in water at RT (mV)	-29.83 ± 1.56	-33.21 ± 2.08
No. of DNA strands/NP	153 ± 18	1328 ± 116
No. of MBA molecules/NP	2412 ± 29	N.A.

Figure S4. Preparation and characterization of core–satellite nanoclusters linked DNA$_{1HL}$/DNA$_{2HL}$. (A) UV-vis spectra of MBA/DNA$_{2HL}$–Au$_{13}$NPs, DNA$_{1HL}$–Au$_{40}$NPs, and hybridized NPs$_{HL}$ suspended in water. (B) Representative TEM image of hybridized NPs$_{HL}$ at the optimal ratio (DNA$_{1HL}$–Au$_{40}$NPs to MBA/DNA$_{2HL}$–Au$_{13}$NPs) of 1:5. The number of surrounding MBA/DNA$_{2HL}$–Au$_{13}$NPs per DNA$_{1HL}$–Au$_{40}$NPs was estimated to be 2.65 ± 0.81, by counting at least 50 DNA$_{1HL}$–Au$_{40}$NP cores in TEM images. (C) Comparison of SERS spectra of core–satellite nanoclusters linked by DNA$_1$/DNA$_2$ and DNA$_{1HL}$/DNA$_{2HL}$. (D) By DLS analysis, MBA/DNA$_{2HL}$–Au$_{13}$NPs and DNA$_{1HL}$–Au$_{40}$NPs had hydrodynamic diameters of 25.28 ± 2.65 and 52.84 ± 3.51 nm, respectively. Their zeta potentials lay between -29 mV and -34 mV. By NanoDrop, the loading of DNA$_{2HL}$ primers on the Au$_{13}$NP core was determined to be 153 ± 18 strands/NP, and the loading of DNA$_{1HL}$ primers on the Au$_{40}$NP core was determined to be 1328 ± 116 strands/NP. By Ellman's assay, the loading of MBA molecules on DNA$_{2HL}$–Au$_{13}$NPs was determined to be 2,412 ± 29 molecules/NP. Numbers in parentheses refer to the PDI. RT = room temperature. N.A. = not applicable. Error bar denotes the standard deviation resulting from three independent experiments.

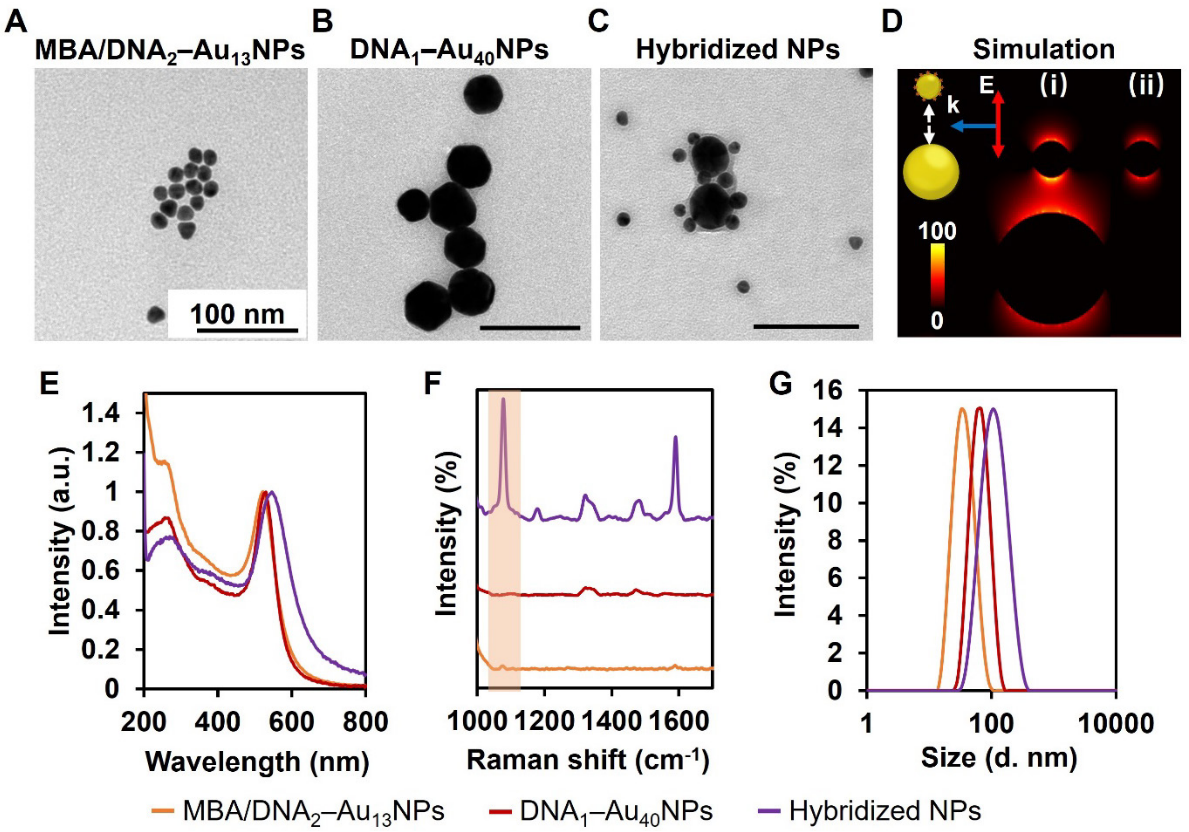

Figure S5. Characterization of hybridized DNA$_1$–Au$_{40}$NPs and MBA/DNA$_2$–Au$_{13}$NPs at an optimal molar ratio of 1:5. Representative TEM images of (A) MBA/DNA$_2$–Au$_{13}$NPs, (B) DNA$_1$–Au$_{40}$NPs, and (C) hybridized NPs. (D) Finite-difference time-domain (FDTD) optical simulation represents one hot spot of the core–satellite nanoclusters. "K" represents the propagation vector of excitation light perpendicular to interparticle separation. "E" represents the electric field of excitation light. (E) The UV-vis spectra of MBA/DNA$_2$–Au$_{13}$NPs and DNA$_1$–Au$_{40}$NPs suspended in water and hybridized NPs suspended in a hybridization buffer. UV-vis spectroscopy shows a redshift in the peak of surface plasmon resonance (SPR) of hybridized NPs at 546 nm when compared to MBA/DNA$_2$–Au$_{13}$NPs and DNA$_1$–Au$_{40}$NPs at 522 and 530 nm, respectively. (F) SERS spectra of MBA/DNA$_2$–Au$_{13}$NPs, DNA$_1$–Au$_{40}$NPs, and hybridized NPs. (G) Size distributions of MBA/DNA$_2$–Au$_{13}$NPs and DNA$_1$–Au$_{40}$NPs suspended in water, and hybridized NPs suspended in hybridization buffer were measured by DLS.

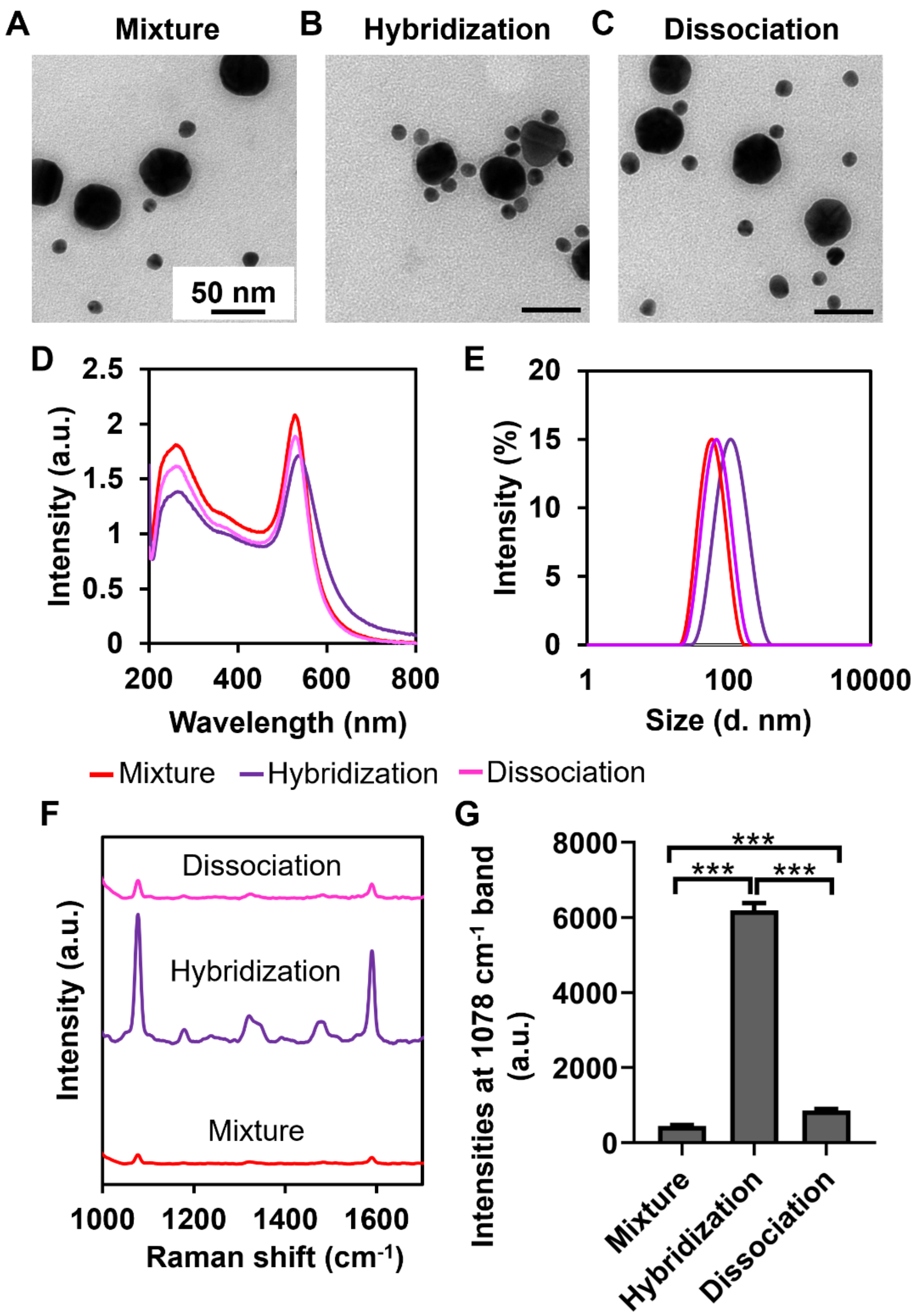

Figure S6. Characterization of DNA_1–Au_{40}NPs and MBA/DNA_2–Au_{13}NPs after mixture, hybridization, and dissociation. Representative TEM images of DNA_1–Au_{40}NPs and MBA/DNA_2–Au_{13}NPs after (A) mixture, (B) hybridization, and (C) dissociation by incubation at a temperature higher than the "melting" temperature (Tm). (D) The UV-vis spectra, (E) size distributions, and (F) SERS spectra of DNA_1–Au_{40}NPs and MBA/DNA_2–Au_{13}NPs after mixture, hybridization, and dissociation. (G) Quantitative analysis of MBA-peak intensities of biosensing platform after detection of samples, corresponding to (F). Error bar denotes the standard deviation resulting from three independent experiments. Statistical analysis of pairwise comparison was determined by one-way ANOVA. *** $P < 0.001$.

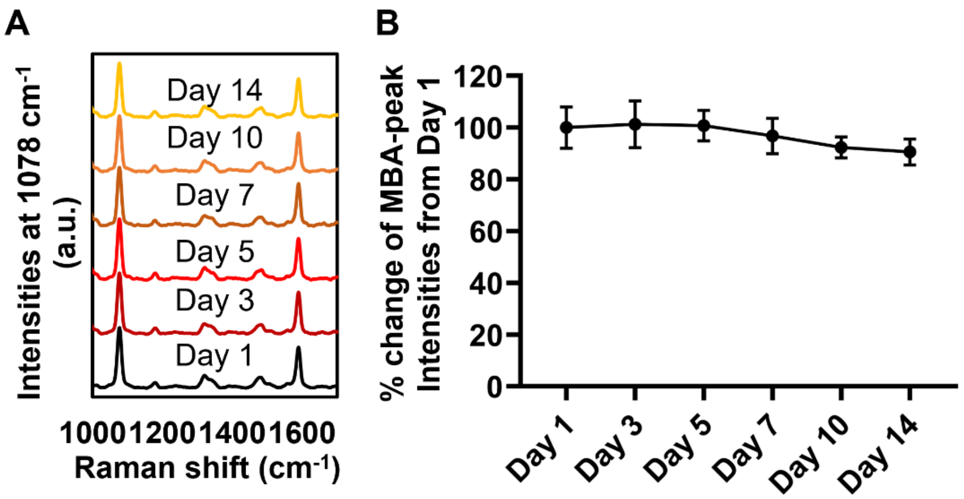

Figure S7. Stability test of core–satellite nanoclusters. (A) SERS spectra of the core–satellite nanoclusters (stored at 4 °C) were measured on Day 1, Day 3, Day 5, Day 7, Day 10, and Day 14. (B) Comparison of MBA-peak intensities of nanoclusters of defined time points, corresponding to (A), with Day 1 group. Error bar denotes the standard deviation resulting from three independent experiments.

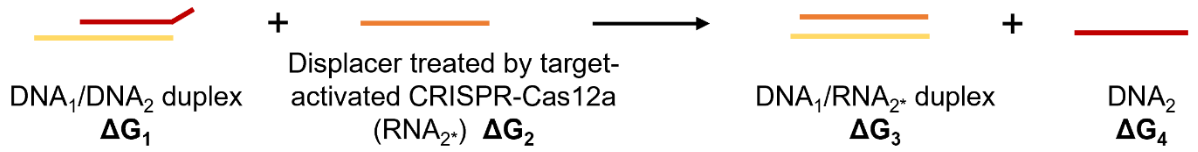

Figure S8. Thermodynamic calculation of toehold-mediated strand displacement reaction TMSDR.

The net equation of the TMSDR is illustrated in order to calculate related thermodynamic parameters. As the treated displacer was destabilized by the CRISPR-Cas12a, RNA_{2*} was released, and hence RNA_{2*} only was considered to participate in TMSDR. According to the definition of Gibbs free energy, the Gibbs free energy change of the reaction is expressed as:

$$\Delta G = \Delta H - T\Delta S \ \ldots \ (1)$$

where ΔH is the change of enthalpy, ΔS is the change of entropy, and T is the thermodynamic temperature. Considering that DNA_1, DNA_2, and RNA_{2*} are originally all single-strand nucleic acids, the base pair in the reactants and the products remain unchanged, giving $\Delta H \approx 0$. The reaction was processed under constant temperature (37 °C). Hence, the reaction is driven forward by the increment of entropy. At any moment of the reaction process, the free energy change is:

$$\Delta G = (\Delta G_3^{\theta} + \Delta G_4^{\theta}) - (\Delta G_1^{\theta} + \Delta G_2^{\theta}) + RT \ln Q \ \ldots \ (2)$$

where Q is the reaction quotient, the superscript "θ" represents the standard conditions (100 mM NaCl, 10 mM phosphate, $c^{\theta} = 1$ M, and pH 7.0 at 37 °C), R represents the ideal gas constant, and T is 310K. ΔG_2^{θ} and ΔG_4^{θ} were calculated from nucleic acid analysis software NUPACK. ΔG_1^{θ} and ΔG_3^{θ} were calculated from the thermodynamic parameters established by

SantaLucia et al. for DNA/DNA duplex stability [7] and Sugimoto et al. for DNA/RNA duplex stability [8], respectively.

$$\Delta G° = (\Delta G_3^{\theta} + \Delta G_4^{\theta}) - (\Delta G_1^{\theta} + \Delta G_2^{\theta}) = -5.49 \text{ kcal/mol}$$

For RNA_{2*} released from 6-nt displacer, when the reaction reaches equilibrium, ΔG = 0, the equilibrium constant K = Q_1. According to equations (2), K is calculated to be 8.42, which is >> 1. With ΔG° < 0 and K > 1, the products are favoured over reactants at equilibrium.

In contrast, the ΔG° between the RNA from the short displacer (1-nt or 3-nt displacer) and DNA_1/DNA_2 was calculated to be 5.01 kcal/mol (K = 0.998, which is < 1) or 0.61 kcal/mol (K = 0.9998, which is < 1), respectively. Hence, 1-nt or 3-nt displacer is not energetically favourable to drive the reaction to the right to reach equilibrium. Therefore, the 6-nt displacer sequence is optimized to mediate the release of DNA_2 via TMSDR.

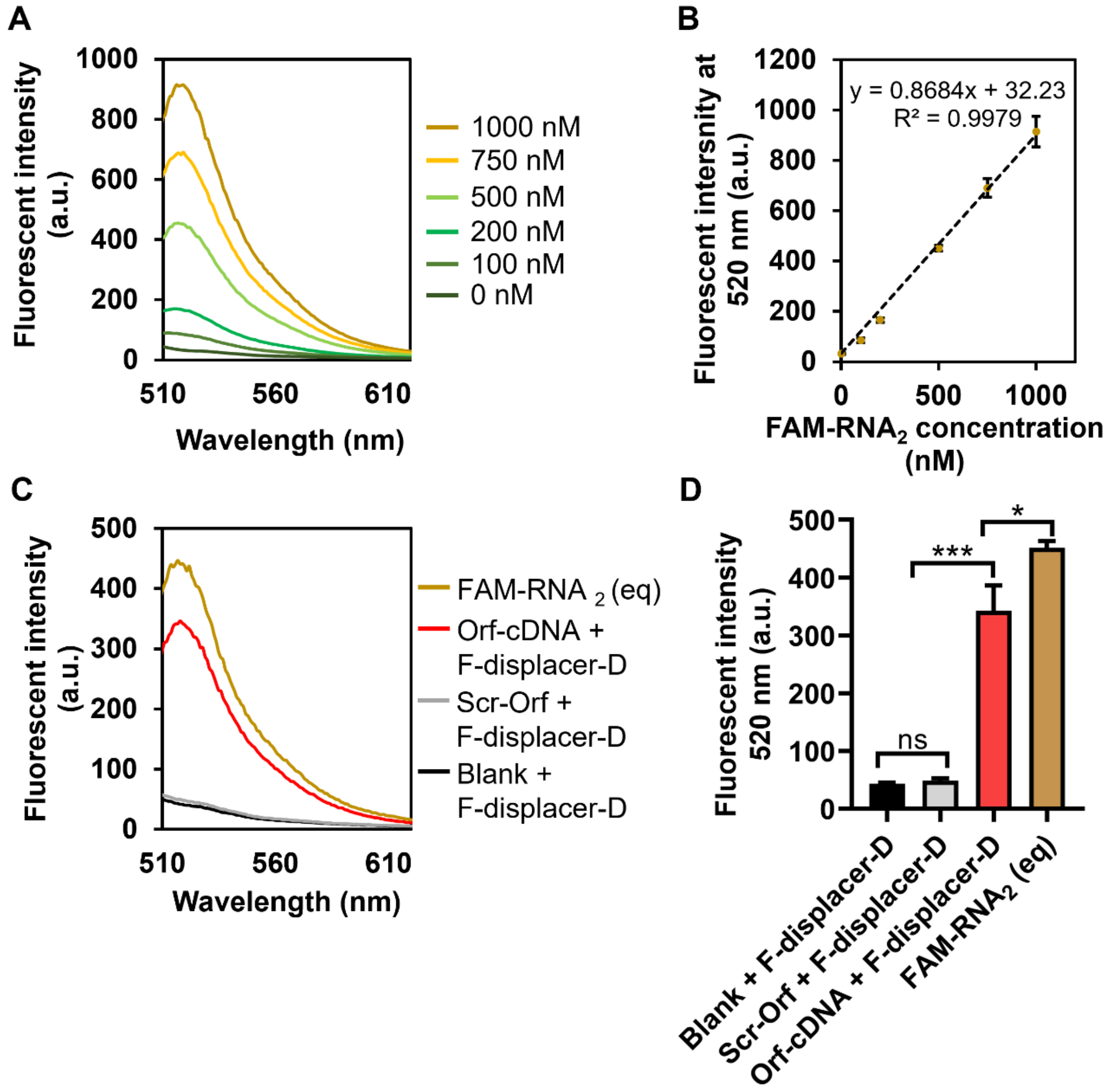

Figure S9. Feasibility study of the release of RNA_{2*} from the displacer after treatment by target-activated CRISPR-Cas12a. (A) Fluorescence spectra of FAM-RNA_2 with various concentrations from 0 nM to 1000 nM, from bottom to top. (B) Fluorescence intensities of FAM-RNA_2 at 520 nm, corresponding to the samples in (A). (C) Fluorescence spectra of

fluorophore (FAM)-displacer-quencher (DABCYL; F-displacer-D) that was incubated with CRISPR-Cas12a/crRNA and the blank samples, Scr-Orf (1 nM), or Orf-cDNA (1 nM), respectively, and an equivalent amount of FAM-RNA_2 to F-displacer-D (500 nM). (D) Fluorescence intensities at 520 nm of samples, corresponding to (C). According to the linear regression in (B), the calculated concentration of released FAM-RNA_{2*} from F-displacer-D (500 nM) after treatment by target-activated CRISPR-Cas12a was 379 nM, indicating 75.9% F-displacer-D was destabilized by target-activated CRISPR-Cas12a and liberated FAM-RNA_{2*}. Error bar denotes the standard deviation resulting from three independent experiments. Statistical analysis of pairwise comparison was determined by one-way ANOVA. No significance (ns): $P > 0.05$; * $P < 0.05$; *** $P < 0.001$.

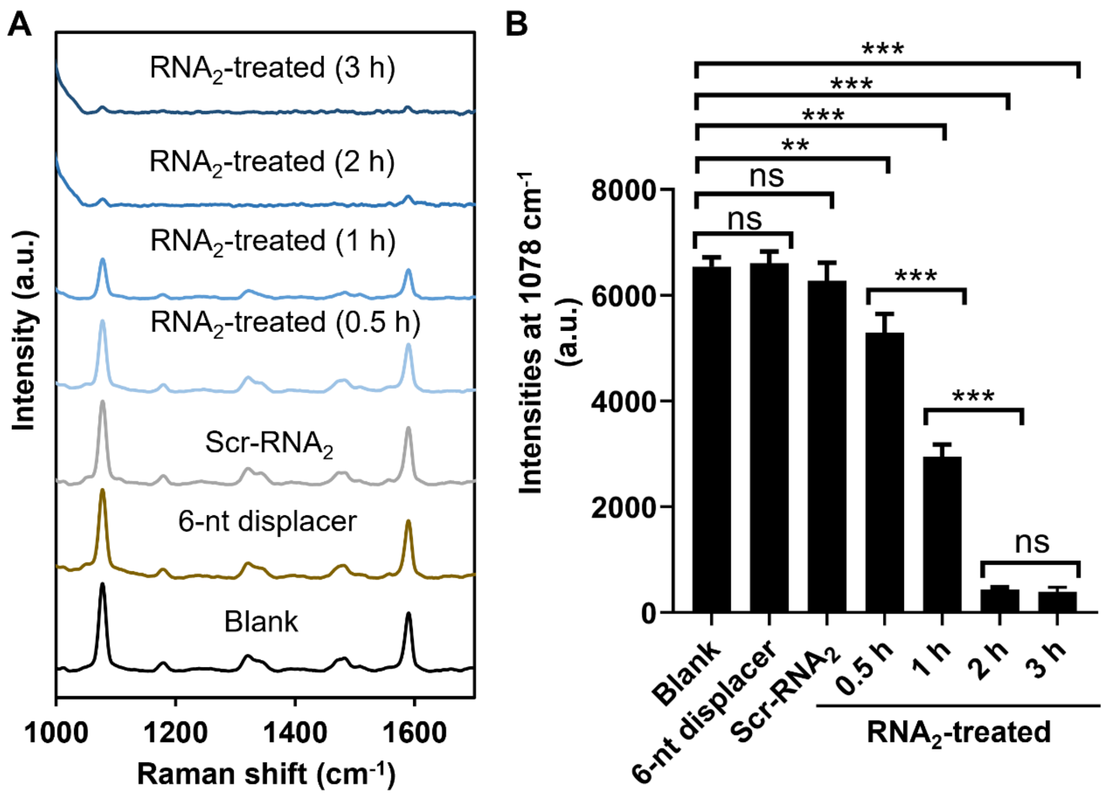

Figure S10. Time-dependent dissociation of hybridized DNA_1–Au_{40}NPs and MBA/DNA_2–Au_{13}NPs by RNA_2. (A) SERS spectra of hybridized NPs after treatment by blank samples, 6-nt displacer, Scr-RNA_2 for 2 h, or RNA_2 for different durations. (B) Quantitative analysis of MBA-peak intensities of biosensing platform after detection of samples, corresponding to (A). Error bar denotes the standard deviation resulting from three independent experiments. Statistical analysis of pairwise comparison was determined by one-way ANOVA. No significance (ns): $P > 0.05$; ** $P < 0.01$; *** $P < 0.001$.

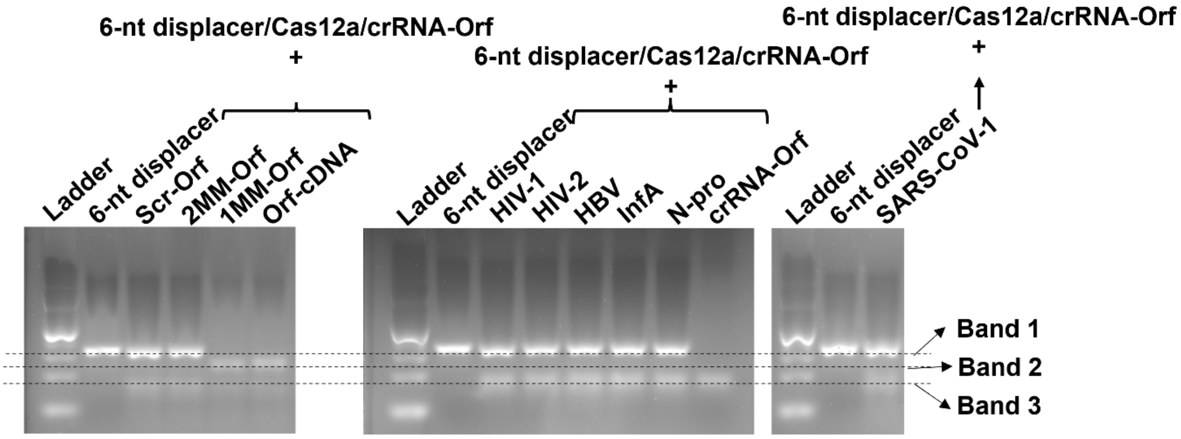

Figure S11. Electrophoresis images of 6-nt displacers after treatment by CRISPR-Cas12a. Electrophoresis image of 6-nt displacer after treatment by CRISPR-Cas12a/crRNA-Orf complex (denoted as 6-nt displacer/Cas12a/crRNA-Orf) in the presence of target Orf-cDNA, its scrambled (Scr-Orf) and mismatched (2MM-Orf and 1MM-Orf) versions, or other nontarget sequences. In the presence of the Orf-cDNA, there was a new and dim band with a noticeable downshift from the position of 6-nt displacers instead of a smeared band, indicating the cleavage of the loop in displacers after 0.5 h of incubation. In contrast, the addition of scrambled or nontarget sequences did not mediate the formation of a new band.

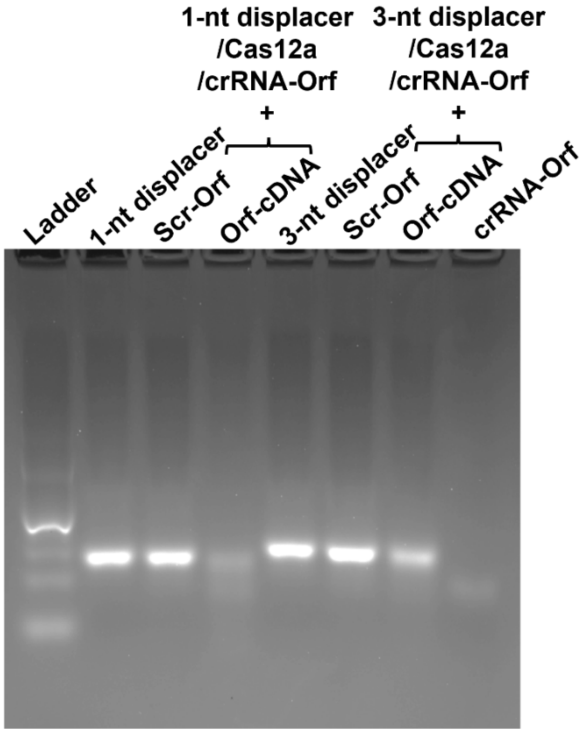

Figure S12. Electrophoresis image of 1-nt or 3-nt displacers after treatment by CRISPR-Cas12a. Electrophoresis image of 1-nt or 3-nt displacer after treatment by CRISPR-Cas12a/crRNA-Orf complex (denoted as 1-nt displacer/Cas12a/crRNA-Orf or 3-nt displacer/Cas12a/crRNA-Orf, respectively) in the presence of target Orf-cDNA or Scr-Orf. In the presence of Orf-cDNA, there was a new and dim band with a noticeable downshift from the position of displacers instead of a smeared band, indicating the cleavage of the loop in displacers after 0.5 h of the incubation.

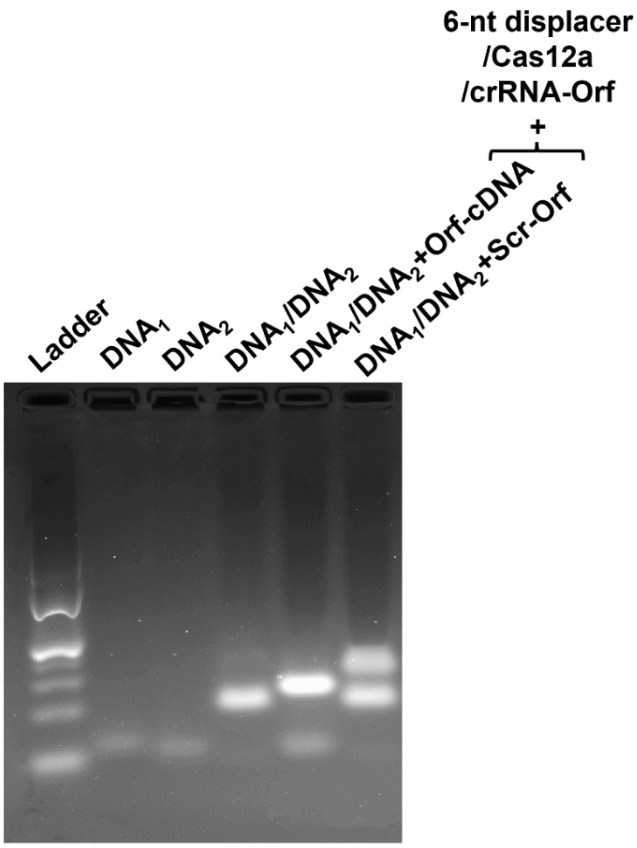

Figure S13. Electrophoresis image of TMSDR mediated by target-activated CRISPR-Cas12a/6-nt displacer. After hybridization, DNA_1/DNA_2 duplex shifted to a higher molecular weight when compared to DNA_1 or DNA_2. After incubating the DNA_1/DNA_2 duplex with the 6-nt displacers that were pre-treated by target-activated CRISPR-Cas12a (DNA_1/DNA_2 + Orf-cDNA + 6-nt displacer/Cas12a/crRNA-Orf), released RNA_{2*} bound to the toehold of DNA_1 and formed a brighter duplex band with a higher molecular weight than that of DNA_1/DNA_2 duplex. Meanwhile, the appearance of DNA_2 band also confirms the TMSDR. In contrast, after incubating the DNA_1/DNA_2 duplex with the 6-nt displacers that were pre-treated by non-activated CRISPR-Cas12a (DNA_1/DNA_2 + Scr-Orf + 6-nt displacer/Cas12a/crRNA-Orf), there were two bands of DNA_1/DNA_2 duplex and 6-nt displacer, without the appearance of DNA_2.

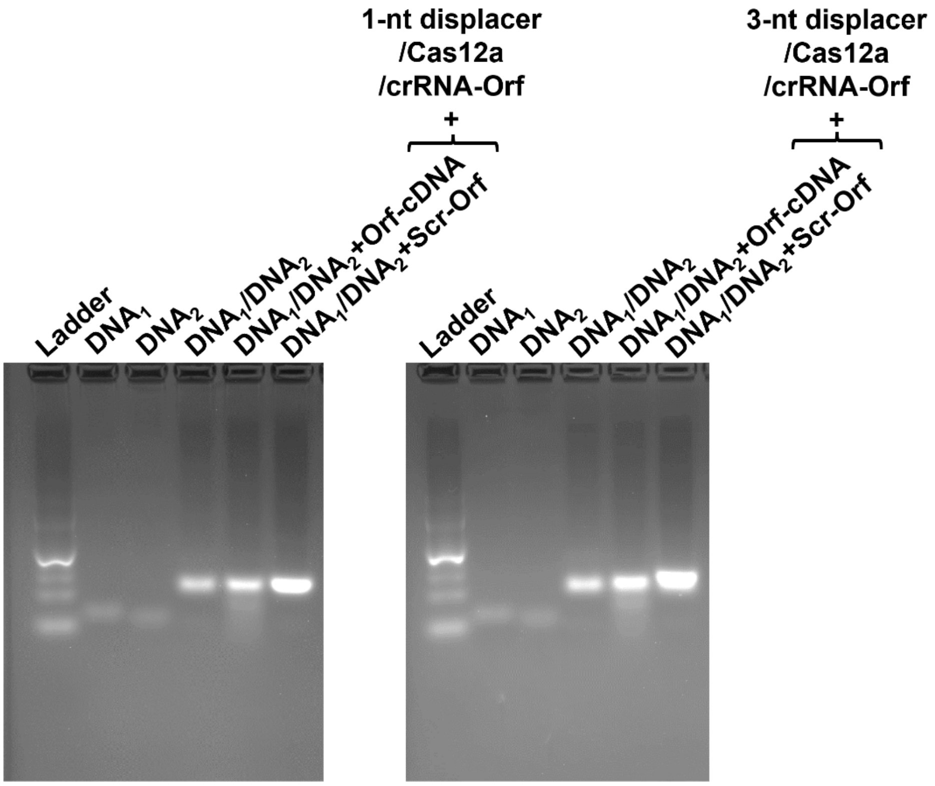

Figure S14. Electrophoresis image of TMSDR mediated by 1-nt or 3-nt displacer/target-activated CRISPR-Cas12a. After hybridization, DNA_1/DNA_2 duplex shifted to a higher molecular weight when compared to DNA_1 or DNA_2. After incubating the DNA_1/DNA_2 duplex with the 1-nt (left) or 3-nt (right) displacers that were pre-treated by target-activated CRISPR-Cas12a (DNA_1/DNA_2 + Orf-cDNA + 1-nt displacer/Cas12a/crRNA-Orf or DNA_1/DNA_2 + Orf-cDNA + 3-nt displacer/Cas12a/crRNA-Orf), there was a brighter duplex band with a molecular weight similar to that of DNA_1/DNA_2 duplex. Also, there was no obvious appearance of DNA_2 band. After incubating the DNA_1/DNA_2 duplex with the 1-nt or 3-nt displacers that were pre-treated by non-activated CRISPR-Cas12a (DNA_1/DNA_2 + Scr-Orf + 1-nt displacer/Cas12a/crRNA-Orf or DNA_1/DNA_2 + Scr-Orf + 3-nt displacer/Cas12a/crRNA-Orf), the displacers merged with DNA_1/DNA_2 duplex (a brighter band). Also, the 3-nt displacer group shifted slightly to a higher molecular weight than the DNA_1/DNA_2 duplex.

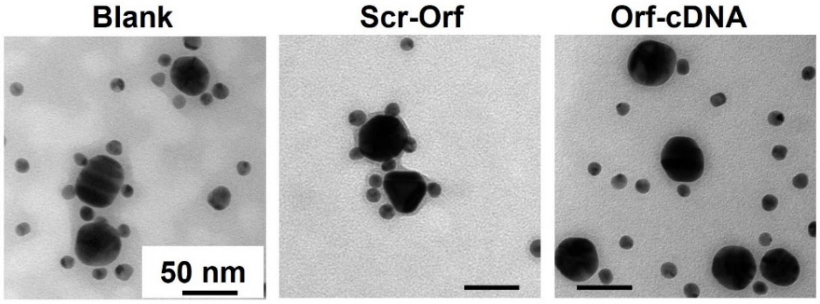

Figure S15. Representative TEM images. Representative TEM images of the core–satellite nanoclusters in the biosensing system after CRISPR-Cas12a/displacer-mediated detection of blank samples, Scr-Orf, and Orf-cDNA at a concentration of 1 nM.

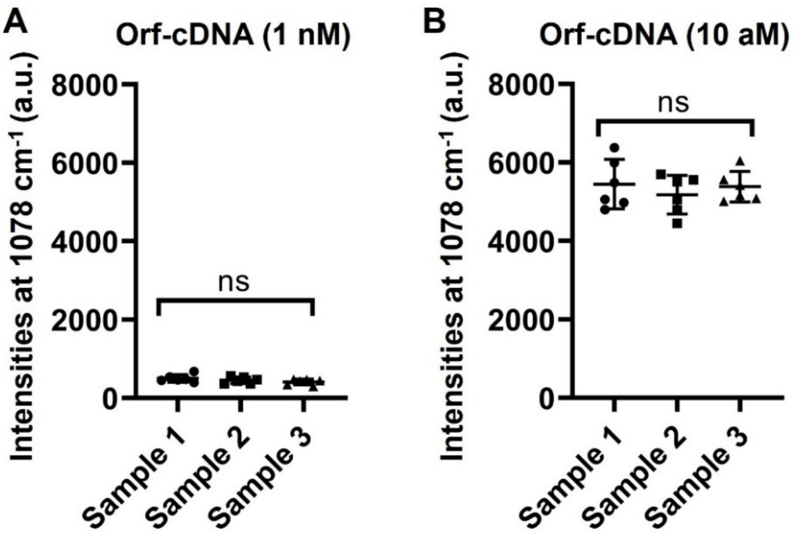

Figure S16. Representative SERS signals collection after detecting Orf-cDNA at 1 nM and 10 aM using the biosensing platform. For each group of concentrations, we examined three independent samples (n=3) and collected SERS signals of six random points from each sample. The resulting SERS signals after detecting (A) 1 nM and (B) 10 aM Orf-cDNA showed the percent relative standard deviation (%RSD) of MBA-peak intensities to be 8.87% and 2.66%, respectively, which indicated good repeatability of our platform. Each dot represents one random point from each sample. Error bar denotes the standard deviation. Statistical analysis of pairwise comparison was determined by one-way ANOVA. No significance (ns): $P > 0.05$.

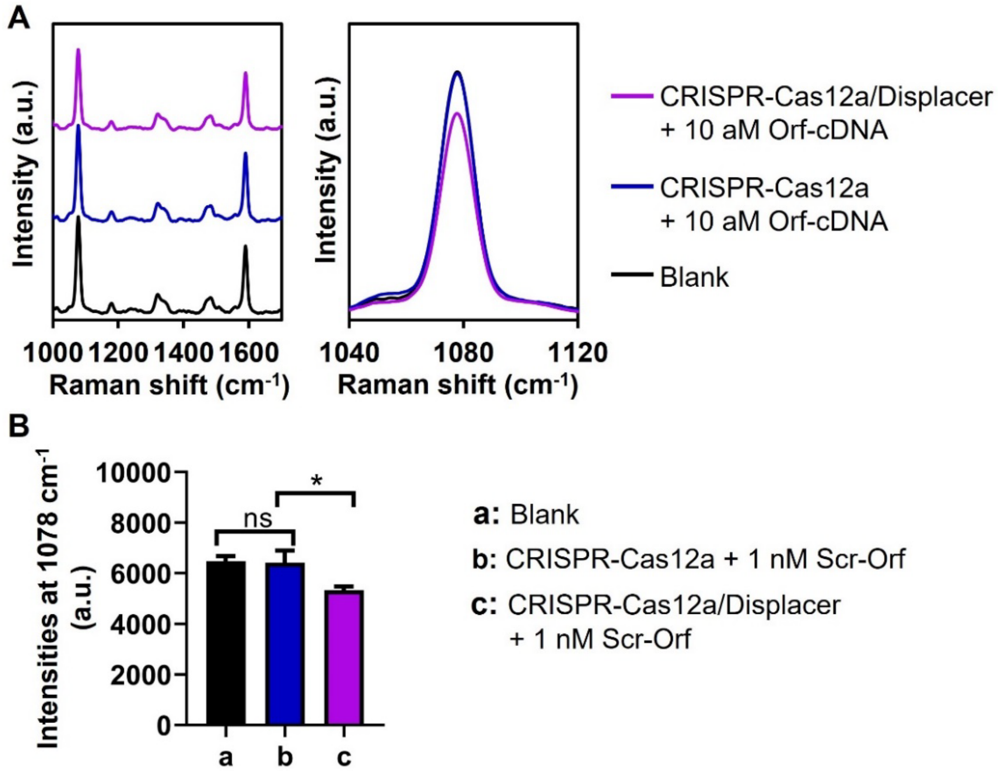

Figure S17. Evaluation of biosensing performance of nanosatellite system by the CRISPR-Cas12a-based method with (CRISPR-Cas12/Displacer) or without displacer

(CRISPR-Cas12a) for detecting Orf-cDNA. (A) SERS spectra of biosensing platform after detecting 10 aM Orf-cDNA by employing CRISPR-Cas12a-based method with or without displacer. SERS spectra were zoomed into the region of 1040 to 1120 cm^{-1} to compare MBA-peak intensities between CRISPR-Cas12a group and CRISPR-Cas12a/Displacer group in the presence of Orf-cDNA at 10 aM. (B) MBA-peak intensities of the biosensing platform after detection of samples, corresponding to (A). Error bar denotes the standard deviation resulting from three independent experiments. Statistical analysis of pairwise comparison was determined by one-way ANOVA. No significance (ns): $P > 0.05$; * $P < 0.05$.

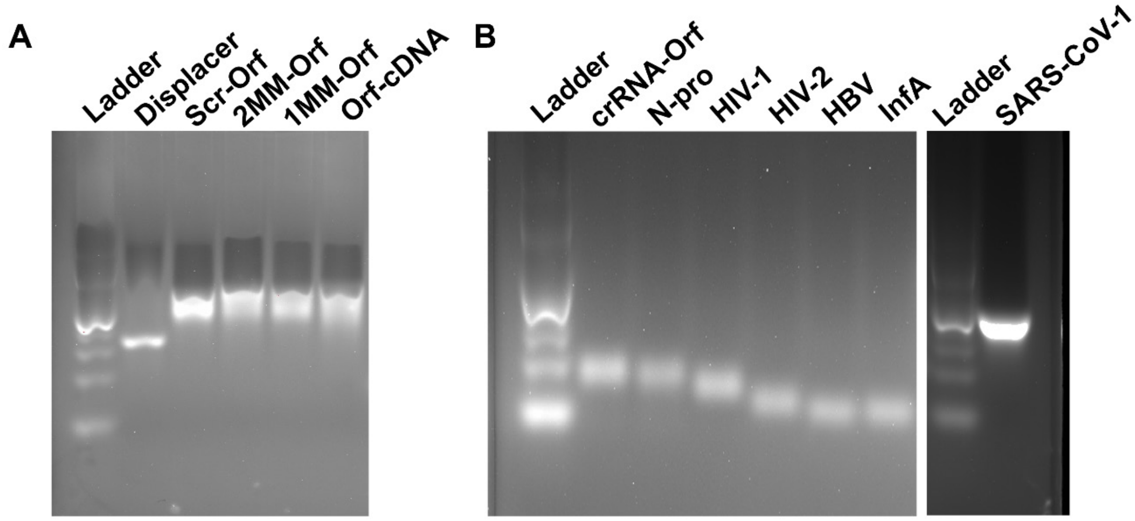

Figure S18. Electrophoresis images. (A) displacer, Orf-cDNA, and its scrambled and mismatched versions. Orf-cDNA has a similar size and molecular weight as its mismatched and scrambled versions presented by the electrophoresis image. (B) crRNA of Orf-cDNA (crRNA-Orf), and six nontarget DNA sequences. The various bands indicate different sizes and molecular weights of the nontargets, corresponding to their sequences listed in Table S1.

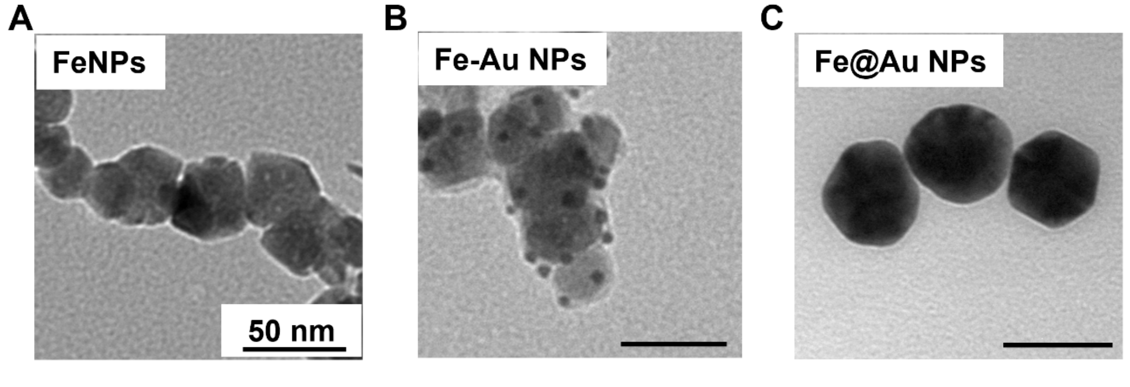

Figure S19. Characterization of magnetic Fe@Au_{40} NPs. Representative TEM images of (A) FeNPs, (B) Fe–Au NPs, and (C) Fe@Au_{40} NPs.

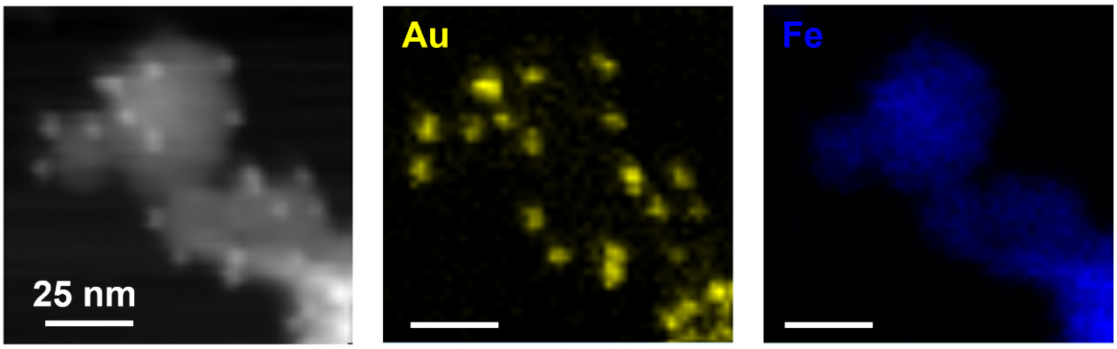

Figure S20. EDX elemental map of Fe–Au NPs. ~5 nm AuNPs were stably adsorbed on FeNP surfaces to mediate seed-growth of Au shell in the next step.

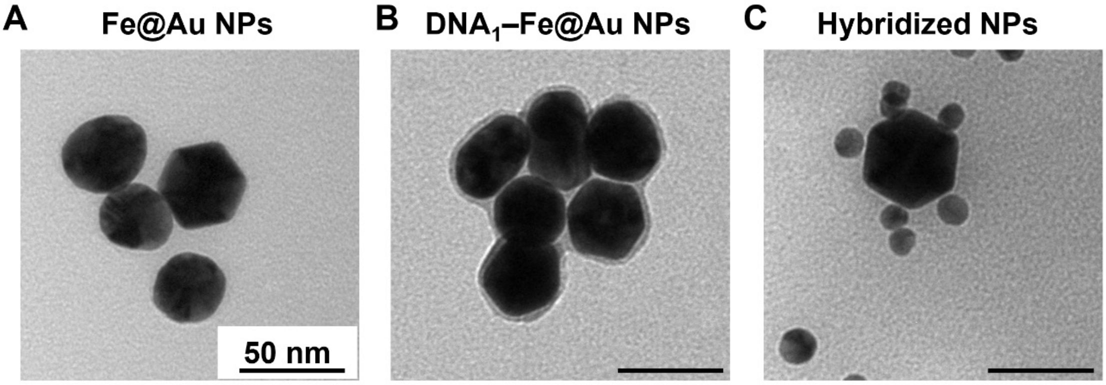

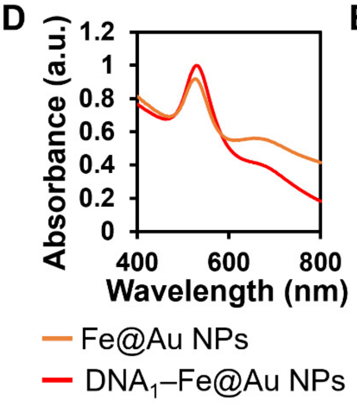

E

Sample	Fe@Au NPs	DNA$_1$–Fe@Au NPs
Hydrodynamic diameter in water at RT (nm)	42.65 ± 0.22 (0.13)	67.36 ± 0.60 (0.25)
ζ-potential in water at RT (mV)	-39.70 ± 1.39	-31.17 ± 0.38
No. of DNA strands/NP	N.A.	1470 ± 134

Figure S21. Characterization of hybridized Fe@Au$_{40}$ NPs and DNA$_1$–Fe@Au$_{40}$ NPs. Representative TEM images of (A) Fe@Au$_{40}$ NPs, (B) DNA$_1$–Fe@Au$_{40}$ NPs, and (C) hybridized DNA$_1$–Fe@Au$_{40}$ NPs and MBA/DNA$_2$–Au$_{13}$NPs. The DNA shell surrounding the Fe@Au$_{40}$ NP core was stained with platinum blue. (D) The UV-vis spectra of Fe@Au$_{40}$ NPs and DNA$_1$–Fe@Au$_{40}$ NPs with their SPR peaks at 528 and 530 nm, respectively. (E) By DLS analysis, Fe@Au NPs and DNA$_1$–Fe@Au$_{40}$ NPs had hydrodynamic diameters of 42.65 ± 0.22 and 67.36 ± 0.60, respectively. Their zeta potentials lay between -30 mV and -40 mV. By NanoDrop, the loading density of DNA$_1$ primers on Fe@Au$_{40}$ NPs was determined to be 1,470 ± 134 strands/NP. Numbers in parentheses refer to the PDI. RT = room temperature. N.A. = not applicable. Error bar denotes the standard deviation resulting from three independent experiments.

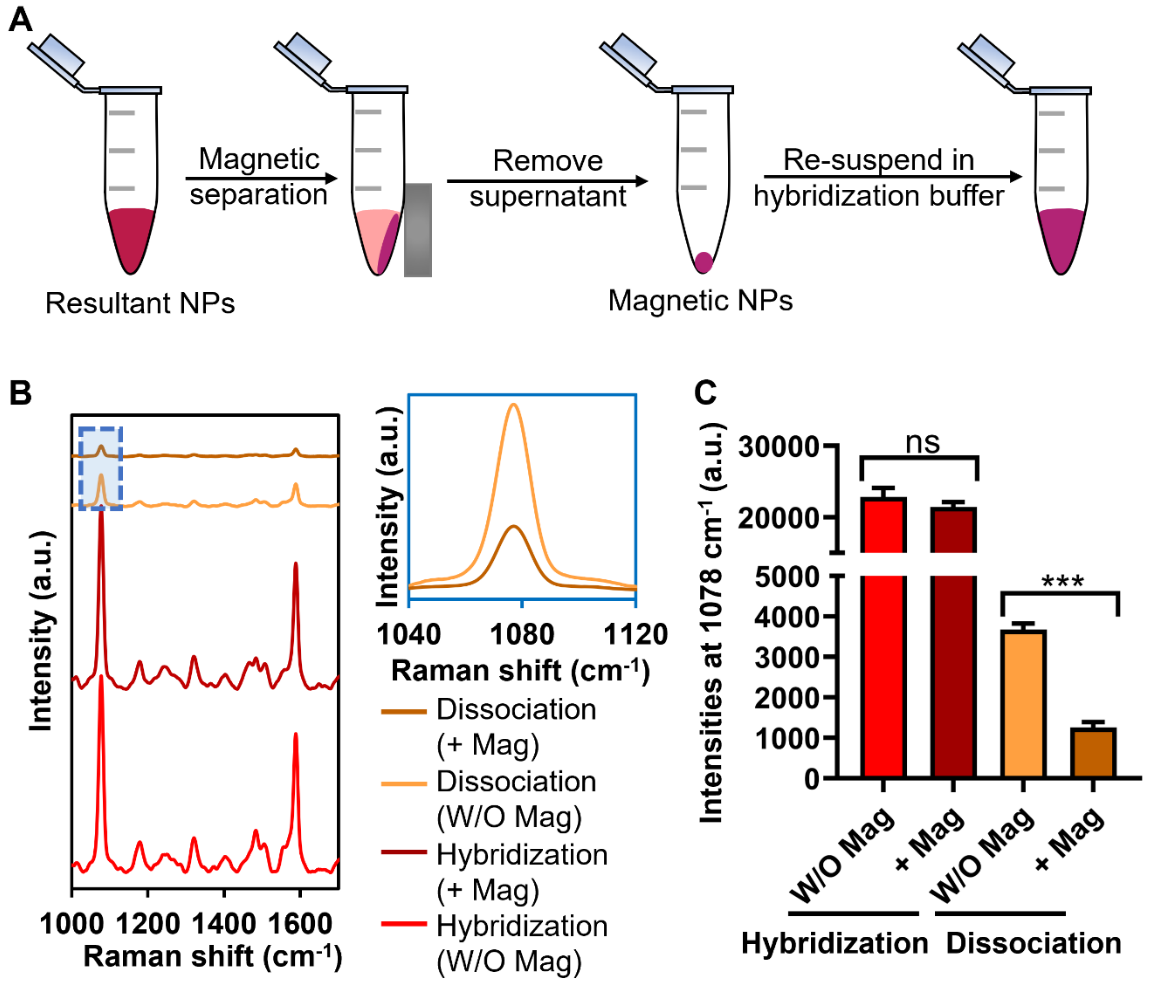

Figure S22. Magnetic separation of hybridized or dissociated (T > Tm) DNA_1–Fe@Au NPs and MBA/DNA_2–Au_{13}NPs. (A) Illustration of magnetic separation of resultant NPs (hybridization or dissociation). Hybridized NPs were dissociated by incubation at 65 ^{o}C (> Tm). Dissociated MBA/DNA_2–Au_{13}NPs without magnetic property were removed after magnetic separation. (B) SERS spectra of hybridized or dissociated NPs with (+ Mag) or without (W/O Mag) magnetic separation. (C) Quantitative analysis of intensities at MBA-peak of samples, corresponding to (B). Error bar denotes the standard deviation resulting from three independent experiments. Statistical analysis of pairwise comparison was determined by one-way ANOVA. No significance (ns): $P > 0.05$; *** $P < 0.001$.

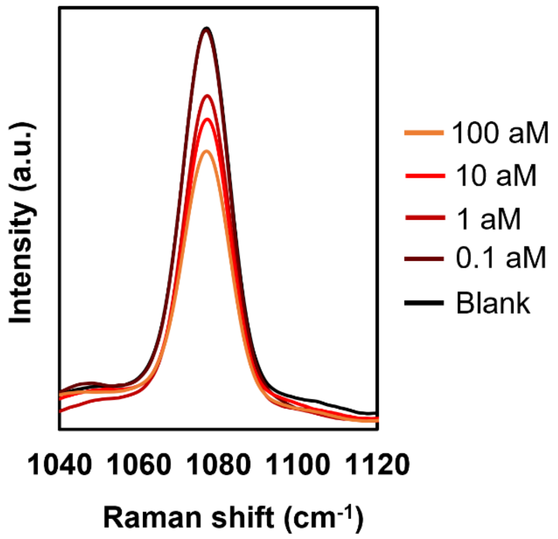

Figure S23. SERS spectra of the magnetic-responsive biosensing platform after detecting Orf-cDNA. Zoom-in image of the MBA-peak intensities of the platforms that were administrated with the blank sample, and Orf-cDNA at varying concentrations (from 0.1 aM to 100 aM), corresponding to Figure 4E in the main text.

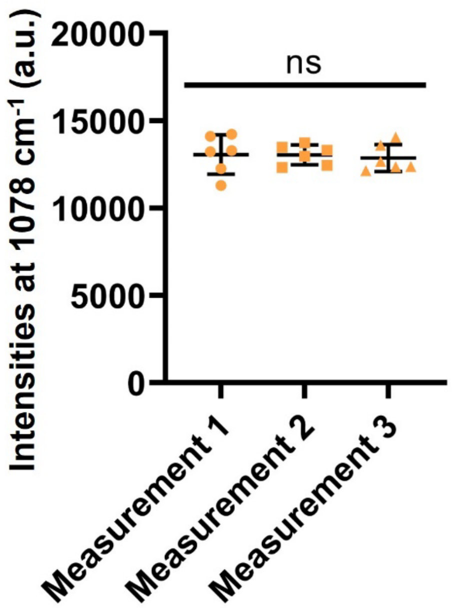

Figure S24. SERS intensities of detecting Orf gene sequence in cell culture-derived viral samples (B.1.1.529/Omicron). Scattering dot diagrams for the MBA-peak intensities were collected from three independent measurements after detecting Orf gene in viral samples. The percent relative standard deviations (%RSD) for Orf gene was 0.86%. Error bar denotes the standard deviation. Each dot represents one random point from each sample. Statistical analysis of pairwise comparison was determined by one-way ANOVA. No significance (ns): $P > 0.05$.

References

1. Choi JH, Lim J, Shin M, Paek SH, Choi JW. CRISPR-Cas12a-based nucleic acid amplification-free DNA biosensor via Au nanoparticle-assisted metal-enhanced fluorescence and colorimetric analysis. Nano Lett. 2021; 21: 693-9.
2. Shan Y, Zhou X, Huang R, Xing D. High-fidelity and rapid quantification of miRNA combining crRNA programmability and CRISPR/Cas13a trans-cleavage activity. Anal Chem. 2019; 91: 5278-85.
3. Fu X, Shi Y, Peng F, Zhou M, Yin Y, Tan Y, et al. Exploring the trans-cleavage activity of CRISPR/Cas12a on gold nanoparticles for stable and sensitive biosensing. Anal Chem. 2021; 93: 4967-74.
4. Dai Y, Somoza RA, Wang L, Welter JF, Li Y, Caplan AI, et al. Exploring the trans-cleavage activity of CRISPR-Cas12a (cpf1) for the development of a universal electrochemical biosensor. Angew Chem Int Ed Engl. 2019; 58: 17399-405.
5. Bruch R, Baaske J, Chatelle C, Meirich M, Madlener S, Weber W, et al. CRISPR/Cas13a-powered electrochemical microfluidic biosensor for nucleic acid amplification-free miRNA diagnostics. Adv Mater. 2019; 31: e1905311.
6. Liang J, Teng P, Xiao W, He G, Song Q, Zhang Y, et al. Application of the amplification-free SERS-based CRISPR/Cas12a platform in the identification of SARS-CoV-2 from clinical samples. J Nanobiotechnology. 2021; 19: 273.
7. SantaLucia J, Jr., Hicks D. The thermodynamics of DNA structural motifs. Annu Rev Biophys Biomol Struct. 2004; 33: 415-40.
8. Sugimoto N, Nakano S, Katoh M, Matsumura A, Nakamuta H, Ohmichi T, et al. Thermodynamic parameters to predict stability of RNA/DNA hybrid duplexes. Biochemistry. 1995; 34: 11211-6.
